# Transcriptome analysis of the cerebral cortex of acrylamide-exposed wild-type and *IL-1β*-knockout mice

**DOI:** 10.1007/s00204-023-03627-9

**Published:** 2023-11-16

**Authors:** Alzahraa Fergany, Cai Zong, Frederick Adams Ekuban, Bin Wu, Satoshi Ueha, Shigeyuki Shichino, Kouji Matsushima, Yoichiro Iwakura, Sahoko Ichihara, Gaku Ichihara

**Affiliations:** 1 Department of Occupational and Environmental Health, Faculty of Pharmaceutical Sciences, Tokyo University of Science, Building No. 15, 2641 Yamazaki, Noda, Chiba 278-8510 Japan; 2 Laboratory of Genetics and Genetic Engineering in Department of Animal Husbandry and Animal Wealth Development, Faculty of Veterinary Medicine, Alexandria University, Alexandria, Egypt; 3 Division of Molecular Regulation of Inflammatory and Immune Diseases, Research Institute for Biomedical Sciences, Tokyo University of Science, Noda, Japan; 4 Division of Experimental Animal Immunology, Research Institute for Biomedical Sciences, Tokyo University of Science, Noda, Japan; 5 Department of Environmental and Preventive Medicine, Jichi Medical University, Shimotsuke, Japan

**Keywords:** Acrylamide, *IL-1β*, Neurotoxicity, Transcriptome, Mouse cerebral cortex

## Abstract

Acrylamide is an environmental electrophile that has been produced in large amounts for many years. There is concern about the adverse health effects of acrylamide exposure due to its widespread industrial use and also presence in commonly consumed foods and others. *IL-1β* is a key cytokine that protects the brain from inflammatory insults, but its role in acrylamide-induced neurotoxicity remains unknown. We reported recently that deletion of *IL-1β* gene exacerbates ACR-induced neurotoxicity in mice. The aim of this study was to identify genes or signaling pathway(s) involved in enhancement of ACR-induced neurotoxicity by *IL-1β* gene deletion or ACR-induced neurotoxicity to generate a hypothesis mechanism explaining ACR-induced neurotoxicity. C57BL/6 J wild-type and *IL-1β* KO mice were exposed to ACR at 0, 12.5, 25 mg/kg by oral gavage for 7 days/week for 4 weeks, followed by extraction of mRNA from mice cerebral cortex for RNA sequence analysis. IL-1β deletion altered the expression of genes involved in extracellular region, including upregulation of PFN1 gene related to amyotrophic lateral sclerosis and increased the expression of the opposite strand of IL-1β. Acrylamide exposure enhanced mitochondria oxidative phosphorylation, synapse and ribosome pathways, and activated various pathways of different neurodegenerative diseases, such as Alzheimer disease, Parkinson disease, Huntington disease, and prion disease. Protein network analysis suggested the involvement of different proteins in related to learning and cognitive function, such as Egr1, Egr2, Fos, Nr4a1, and Btg2. Our results identified possible pathways involved in IL-1β deletion-potentiated and ACR-induced neurotoxicity in mice.

**Supplementary Information:**

The online version contains supplementary material available at 10.1007/s00204-023-03627-9.

## Introduction

Acrylamide (ACR) has been used extensively in paper and textile industries, as well as for soil conditioning, wastewater treatment, and polymer production (Smith and Oehme 1991). In 1994, ACR was listed as a class 2A substance by the International Agency for Research on Cancer (IARC) (Zhang and Zhang 2007). In 2002, the finding of ACR in various cooked foods and the mechanism of ACR formation through the Maillard reaction was reported (Mottram et al. 2002; Stadler et al. 2002). Current evidence suggests that exposure to ACR is associated with selective neurotoxicity in humans (LoPachin and Gavin 2012). Case reports suggested the major clinical features of human ACR intoxication are related to polyneuropathy, including ataxia, skeletal muscles weakness, and numbness of the extremities (Auld and Bedwell 1967), as well as damage of the central nervous system. More recently, experimental studies demonstrated that exposure to ACR-induced peripheral and central nerve fiber degeneration, which occurs first in the extremities of long and large nerve fibers (LoPachin et al. 2003). LoPachin and colleagues proposed that the nerve terminals were the primary site of ACR action, and that ACR-induced neurotoxicity was based on impaired synaptic transmission in peripheral nerves and the central nervous system due to ACR-induced formation of adducts with cysteine residues on specific proteins involved in synaptic transmission (LoPachin et al. 2003; LoPachin 2004; LoPachin and Gavin 2012). Their studies suggested that these changes ultimately lead to dysfunction and neuronal degeneration (LoPachin 2004).

On the other hand, the roles of glial cells and inflammatory signals in ACR-induced neurotoxicity remains elusive at this stage. Glial cells, such as astrocytes and microglia, play an important role in brain function, including neurotrophic support, transporter regulation, pathogen elimination, induction of neuronal differentiation, and modulation of the immune response (Anderson and Swanson 2000; Streit et al. 1988). When activated by injury or infection, glial cells are known to secrete various neurotoxic signals, such as reactive oxygen species (ROS) and proinflammatory mediators, such as cytokines (Li et al. 2014; Sloan and Barres 2014). Furthermore, glial cells play an important role in the onset and progression of neurotoxicity and various brain pathologies (Dheen et al. 2007). The neuroinflammatory response following CNS injury can be either harmful or paradoxically beneficial (Szalay et al. 2016; Ransohoff et al. 2015), but the role of inflammation in the progression of degeneration and regeneration following CNS trauma remains elusive (Mietto et al. 2015). IL-1β is secreted in the brain by activated astroglia and microglia, where it has a wide range of effects on immune function and coordination of various aspects of the acute phase response to trauma and infection (Murray et al. 2015; John et al. 2005). It is widely accepted that the inflammatory processes stimulated by *IL-1β* are harmful and can exacerbate the primary damage caused by CNS infections (Medel-Matus et al. 2014). Overexpression of IL-1β has been identified in various inflammatory and degenerative CNS conditions (Silva et al. 2015; Hopkins and Rothwell 1995). Although proinflammatory cytokines are thought to be important mediators of neuroinflammation, their role in the case of brain injury is unidentified.

We reported previously microglial activation and upregulation of proinflammatory cytokines, including IL-1b, in ACR-induced degeneration of monoaminergic axons both in in vivo and in vitro experiments (Zong et al. 2019). Following that study, we demonstrated that the deletion of *IL-1β* potentiated the ACR-induced increase in landing foot spread, which serves as a marker of motor dysfunction (Fergany et al. 2023). Additionally, *IL-1β* deletion exacerbated the ACR-induced decrease in the density of noradrenergic (NA) axons in the somatosensory cortex area. These neurological changes were accompanied by specific alterations in oxidative stress parameters. IL-1β deletion suppressed the ACR-induced increase in both total and oxidized glutathione levels. Furthermore, IL-1β deletion resulted in the suppression of ACR-induced upregulation of antioxidant genes, including Gpx1, Gpx4, and Gclc. Conversely, there was a downregulation of these antioxidant genes in IL-1β knockout (KO) mice compared to their wild-type counterparts. These findings emphasize the significant role of IL-1β in modulating motor dysfunction, axon density, and oxidative stress responses triggered by ACR exposure.

The present study is an extension to the above studies and involved transcriptome analysis to understand the molecular mechanism of the protective effect of proinflammatory cytokine *IL-1β* in ACR-induced neurotoxicity of mouse brain.

## Materials and methods

### Chemicals and preparation

Acrylamide was purchased from Sigma–Aldrich (lot #A9099, purity > 99%, St. Louis, MO). It was freshly prepared at the start of each week by dissolving in drinking water filtered through a G-10 ion exchange cartridge (Organo Co., Tokyo, Japan), stored at 4 °C and administered each day in autoclaved tubes by oral gavage.

### Animal husbandry and experimental design

A total of 18 IL-1β-knockout mice (C57BL/6msSlc background purchased from SLC Japan, Inc., Hamamatsu, Japan) were produced and used in the study at 10 weeks of age. The IL-1β KO mice (Horai et al. 1998) were backcrossed C57BL/6msSlc having a congenicity of > 99.998 at the Institute of Medical Science, the University of Tokyo. At 6–8 weeks of age, the DNA was extracted from ear samples obtained from each mouse and analyzed by polymerase chain reaction (PCR) to confirm its genotype using primers (Lac Z GAGGTGCTGTTTCTGGTCTTCACC, *IL-1β* common CACATATCCAGCACTCTGCTTTCAG, *IL-1β* W TGGTCAGTGTGTGGGTTGCCTT). The PCR was conducted by a three-step cycle under conditions of 96 °C for 2 min followed by 35 cycles of 96 °C for 20 s, 59 °C for 30 s and 72 °C for 45 s. The amplified DNA samples were then run on a 2% agarose gel electrophoresis and visualized by a CCD camera (Fusion Solo S, Vilber Lourmat, Collegien, France). *IL-1β* KO mice (−/−) showed one band (600 bp) which confirmed that all the mice were homozygous recessive. Specific pathogen-free age matched male C57BL/6msSlc wild-type control mice (*n* = 18) were purchased from SLC Japan, Inc. (Hamamatsu) and acclimatized to the new environment for 1 week before the start of treatment or toxicity studies. All mice were initially housed in separate cages of 4–6 and had access to filtered drinking water and normal chow diet (Charles River Formular-1; 5LR1) ad libitum. They were housed in a controlled environment of temperature (23–25 °C), humidity (57–60%), and light (lights on 0800 h, off 2000 h). After the acclimatization period, the mouse was weighed first and then assigned at random to one of six groups, each consisting of 10 mice, which were exposed to acrylamide (0, 12.5 or 25 mg/kg). Groups 1 to 3 (wild-type mice) and groups 4–6 (*IL-1 β* KO mice) were exposed to acrylamide. Acrylamide was dissolved in drinking water filtered through G-10 ion exchange cartridge (Organo Co.) and administered by oral gavage. Mice of each group (*n* = 6 each) were housed six per cage for morphology and biochemical analysis and treated with the compounds every day of the week for 4 weeks. In the present study, 25 mg/kg was used as the highest exposure level for acrylamide based on the findings of previous studies in rats using 20 mg/kg body weight (Zong et al. 2019).

The study protocol and experimental design were approved by the animal experiment committee of the Tokyo University of Science (Experiment approval Number Y 21016) and strictly followed the guidelines of Tokyo University of Science on animal experiments in accordance with the Japanese act on welfare and management of animals.

### Isolation of total mRNA

Total messenger RNA (mRNA) was isolated from the cerebral cortex (*n* = 6 per group) using the ReliaPrep™ RNA Tissue Miniprep System (Promega, Madison, WI) and the instructions provided by the manufacturer. The concentration of the extracted mRNA following elution with RNase-free water was measured using a NanoDrop 2000 spectrophotometer (Thermo Fisher Scientific, Waltham, MA). The quality of mRNA was determined by confirming that the A260/A280 ratio was ≥ 2.0 after measuring absorbance at 260 nm and 280 nm.

### Preparation of bulk-RNA sequencing library

Transcriptome libraries were prepared from RNA samples harvested from the cerebral cortex of both the wild-type and *IL-1β* KO mice. PolyA RNAs were isolated using Dynabeads M-270 Streptavidin (Thermo Fisher Scientific) conjugated with biotin-3’ WTA-EcoP-dT25, according to the method described previously with some modifications (GSE110711) (Shichino et al. 2019; Aoki et al. 2021). To perform reverse transcription, beads were suspended in 10 μL of RT mix [5 × Superscript IV buffer (Thermo Fisher Scientific), 1 mM dNTP (Roche), 5 mM DTT (Thermo Fisher Scientific), 1 M betaine (Sigma), 6 mM MgCl2, 1 U/μL RNaseIn Plus Rnase Inhibitor (Promega, Madison, WI), and 10 U/μL Superscript IV (Thermo Fisher Scientific)], and then incubated for 5 min at 35 °C, 30 min at 42 °C, and immediately cooled on ice. The beads were washed once with B&W-T buffer [5 mM Tris–HCl (pH 8.0) (Nippon Gene, Tokyo), 1 M NaCl (Merck), 1 mM EDTA (Nippon Gene) and 0.05% Tween-20 (Merck)], and once with Tris–HCl (pH 8.0). To digest free-primer, beads were then suspended in 10 μL of Exo I mix [10 × Exo I buffer (New England Biolabs, Ipswich, MA), 2 U/μL Exonuclease I (New England Biolabs)], and incubated for 30 min at 37 °C and immediately cooled on ice. The beads were washed twice with B&W-T buffer, and once with Tris–HCl (pH 8.0). To add polyC tail, the beads were then suspended in 10 μL of polyC tailing mix [10 × Thermopol buffer (New England Biolabs), 2 mM dCTP (Roche), 0.1 mM ddCTP (GE Healthcare), 1 mM CoCl_2_ (Roche), RNaseH (Invitrogen), and 15 U/μL TdT enzyme (Roche)], and incubated for 30 min at 37 °C, followed by immediate cooling on ice. The beads were washed once with B&W–T buffer, and once with Tris–HCl (pH 8.0). To synthesize the second strand, the beads were suspended in 10 μL of 2nd strand mix [1 × KAPA Hifi Hotstart ReadyMix (KAPA Biosystems, Wilmington, MA), 0.4 μM of primer (illumina-i7-9G)], and the thermal cycling was performed under the following condition: 3 min at 95 °C, 20 s at 98 °C, 16 cycles of 1 min at 47 °C and 2 min at 72 °C, followed by 5 min at 72 °C, and hold at 4 °C. To amplify the total cDNA, 10 μL of the cDNA-containing beads were added with 15 μL of the first PCR mix [0.32 μM of primer (illumina-i7), 0.48 μM of primer (NH2-3’ WTA), and 1× KAPA Hifi Hotstart ReadyMix], and the thermal cycling was performed under the following condition: 3 min at 95 °C, 12 cycles of 20 s at 98 °C, 15 s at 65 °C, and 5 min at 72 °C, followed by 5 min at 72 °C, and hold at 4 °C. The first PCR products were purified by AmPure XP beads (Beckman-Coulter) at 0.6:1 ratio of reagents to sample and eluted with 25 μL of nuclease-free water. Then, 6.3 mL of the purified first PCR product was mixed with the 8.7 μL of the second PCR mix [0.4 μM of primers (illumina-i7, NH2-3’ WTA), and 1× KAPA Hifi Hotstart ReadyMix], and the thermal cycling was performed as follows: 3 min at 95 °C, 5 cycles of 20 s at 98 °C, 15 s at 65 °C, and 5 min at 72 °C, followed by 5 min at 72 °C, and hold at 4 °C. The second PCR products were purified by AmPure XP beads at 0.6:1 ratio of reagent to sample and eluted with 15 μL of 10 mM Tris–HCl (pH 8.0). Furthermore, 100 ng of the whole-transcriptome library was subjected to fragmentation/end-repair/A-tailing using NEBNext Ultra II FS DNA Library Prep Kit for Illumina (New England Biolabs) with some modifications. The thermal cycling was performed as follows: 7 min at 32 °C, 30 min at 65 °C, and hold at 4 °C. Then, 1.25 μL of 1.5 μM illumine adapter was used for adapter ligation. The ligated products were purified by double size selection with 0.4× → 0.7× (final 1.1×) AmPure XP beads and eluted with 10 μL of nuclease-free water. The barcoding PCR was performed with 22.5 µL of barcoding mix [7.5 μL of the resulted eluates, 1 μM primers (ILMN_[UDI]_i5 and ILMN_[UDI]_i7), and 1× NEBNext Ultra II Q5 (New England Biolabs)], and the thermal cycling was performed as follows: 30 s at 98 °C, 9 cycles of 10 s at 98 °C, and 75 s at 65 °C, followed by 5 min at 65 °C, and hold at 4 °C. The resultant products were purified twice by double size selection with 0.5× → 0.8× (final 1.3×) AmPure XP beads and elution with 12 μL of 10 mM Tris–HCl, pH 8.0. The size distribution of the amplified products was analyzed by the MultiNA system (Shimazu, Kyoto, Japan) at appropriate dilutions. Final transcriptome libraries, with lengths around 300 base pairs, were quantified using the KAPA Library Quantification Kit (KAPA Biosystems). The pooled libraries were sequenced by Illumina Novaseq 6000 sequencer (Illumina, San Diego, CA).

### Analysis of bulk-RNA sequencing data

Adapter trimming and quality filtering of the sequencing data were performed by using Cutadpat-v2.10. The filtered reads were mapped to a reference RNA (GRCm38 release-101), using Bowtie2-2.4.2 (parameters: -p 2-L 16-very-sensitive-local-N 1-nofw-seed 656565-reorder) and the read numbers of each gene were counted. The transcriptome data were analyzed as described previously (Shichino et al. 2019). Briefly, between-sample normalization was performed against raw count data using the R 3.5.1. (https://cran.r-project.org/) and TCC package (EEE-E method) (Sun et al. 2013; Tang et al. 2015). Genes with the adjusted *P* < 0.05, fold change ≥ 2, and maximum expression ≥ 100 were identified as statistically significant DEGs. The raw data generated from the experiment have been deposited in the NCBI Gene Expression Omnibus (GEO, http://www.ncbi.nlm.nih.gov/geo), gene bank accession number (GSE211746). Co expressed gene modules among the DEGs were detected by using WGCNA package version 1.71 (Langfelder and Horvath 2008). Functional analysis of the gene module groups was performed using David software. Pathway enrichment analysis was performed on unique ACR-induced up- and down-regulated DEGs in wild-type and *IL-1β* KO mice in the cerebral cortex, using David software. Significantly enriched GO terms (Ashburner et al. 2000) (GO biological process, GO levels 3–8, version: 2021/5/1) and Kyoto Encyclopedia of Genes and Genomes (KEGG, version: 2020/8/5) pathway terms (Kanehisa and Goto 2000) in gene module groups were explored and grouped, and a term network was constructed based on the overlap of their elements using the default software setting. Leading terms within each group were defined as the most significantly enriched term in each group. DEGs were converted to Mouse Ensemble IDs (species: Mus musculus), which were used as the input gene list. The analyses included the selected canonical pathway databases: GO Biological process and KEGG. Differentially expressed genes in the mice cerebral cortex that were unique to wild-type and *IL-1β* KO mice treated with different doses of ACR (0, 12.5, 25 mg/kg) were identified for both upregulated and downregulated DEGs. Gene Ontology (GO) analysis was performed on unique up- and down-regulated DEGs using David software (https://david.ncifcrf.gov/). GO biological process terms were identified using a Bengimin Hoschet and a false discovery rate of 0.05 (Ashburner et al. 2000; Mi et al. 2017).

### Functional protein association network

The protein network for each module was analyzed using STRING database website (https://string-db.org/) by inputting the list of genes into the STRING database, followed by calculating the network, visualizing the network and calculating the degree by cytoHubba plugin using Cytoscape version (3.9.1) software.

## Results

**Fig. 1:**
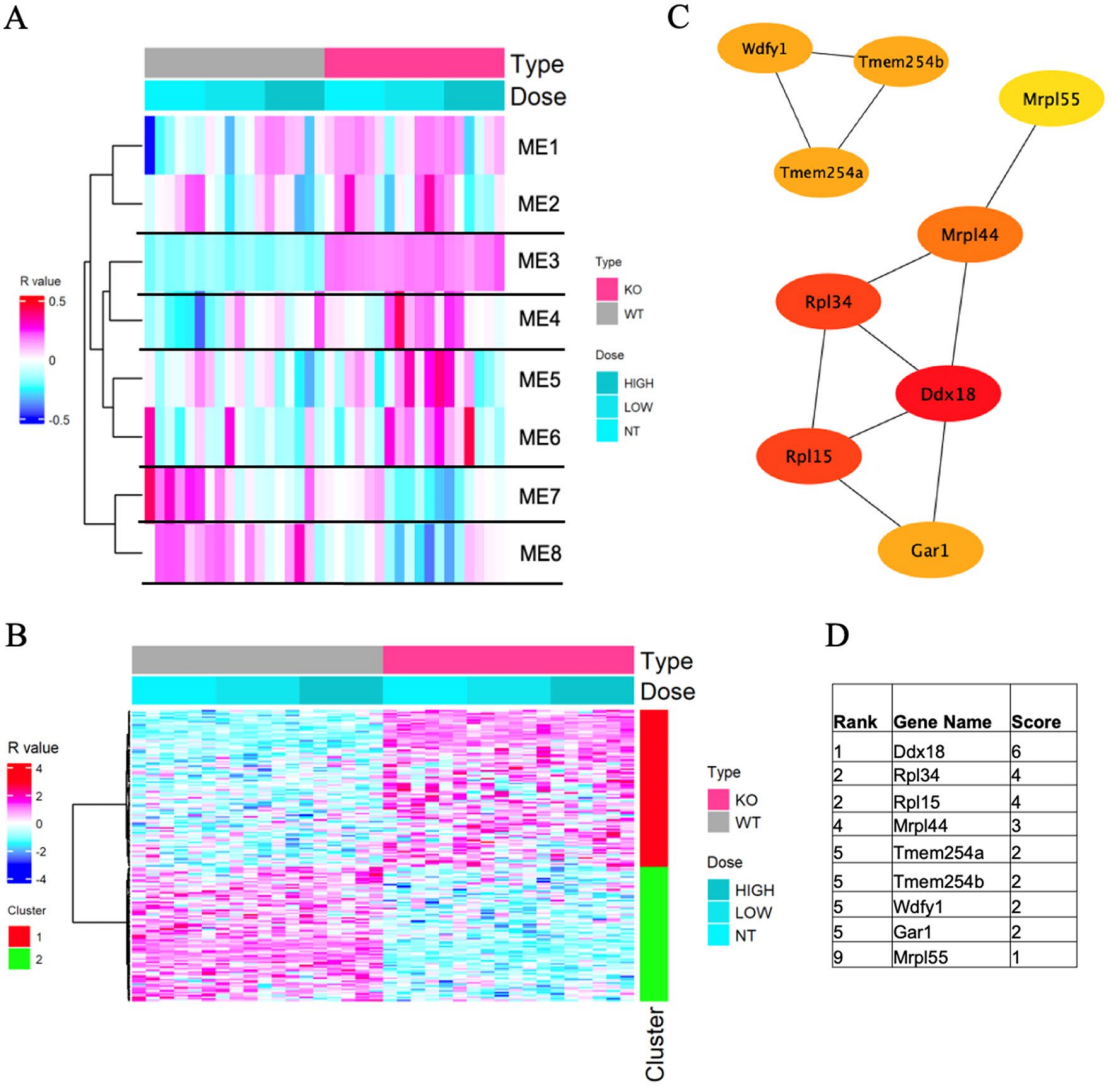
**A** Heatmap of co-expressed gene modules identified by the weight co-expressed network analysis of wild-type (WT) and *IL-1ß* KO mice. *Columns*: acrylamide exposure groups (0, 12.5, 25 mg/kg) for 28 days of both WT and *IL-1ß* KO mice. Rows: individual module eigengene. **B** Heatmap of module eigengene 3 (ME3, 184 genes) of the cerebral cortex of acrylamide-exposed WT and IL-1ß KO mice. Columns: acrylamide exposure groups at 0, 12.5, 25 mg/kg. Red cluster (1): upregulated genes, green cluster (2): downregulated genes in *IL-1ß* KO mice compared to WP mice. **C** Protein network analysis of ME3 module using STRING software. **D** Table shows the scores of the top proteins in the ME3 arranged from the highest score (top) to the lowest score (bottom) (color figure online)

RNA sequence analysis was carried out to determine the effects of *IL-1β* deletion and exposure to ACR on the gene expression in the cerebral cortex. The analysis identified 2187 DEGs with adjusted *P* values < 0.05 (*q* value); fold change of ≥ 2 between at least two samples; and maximum expression of ≥ 100, compared with eight modules by WGCNA (Fig. 1A).

Bulk RNA transcriptome analysis of the *IL-1β* KO mice brain showed increased expression of the IL-1b antisense strand (IL1bos, FC = 3.39) (Supplementary Table 1). In module eigengene 3 (ME3) (Fig. 1B), which provides comparison of the two genotypes (wild-type and *IL-1β* KO mice), overexpression was identified by David software analysis in 184 genes listed in the GO and KEGG. The analysis showed overexpression of the GO: 0005576-extracellular region (*q* = 0.0070) (Table 1), with significant upregulation of the SNORC (*q* = 2.05E-07), PFN1 (*q* = 2.81E−17), CRHBP (*q* = 6.21E−09), PARM1 (*q* = 0.002867) genes, and significant downregulation of the LY86 (*q* = 5.84E−27), MYOC (*q* = 5.77E−08) and NMI (*q* = 0.000248) genes (Table 2). Protein network analysis of module ME3 that included genes with a *q* value of < 0.05 showed the highest scores for Ddx18, RPL15, RPL34, Mrpl44 (Fig. 1C, D).

**Table 1:** GO (biological process) and KEGG pathways for different modules of transcriptome analysis in wild-type and IL-1b KO mice exposed to ACR at 0, 12.5, or 25 mg/kg for 28 days by oral gavage

Category	Term	Module	Count	*p* value	*q* value (*p* value)*	Genes
GOTERM_CC_DIRECT	GO: 0005576 ~ extracellular region	ME3	25	3.43E−05	0.007023595	TNFAIP6, HMGB2, THBS2, ISM1, THBS4, FSTL5, CRHBP, PNP, ADAMTS13, DPP7, REG2, PTGDS, PAMR1, LAG3, PLA2G12A, MYOC, COL25A1, IL15, LY86, RNASE4, NMI, SNORC, PFN1, PAM, HBEGF
KEGG_PATHWAY	mmu05012: Parkinson disease	ME4	16	3.18E−05	0.007536264	NDUFB8, NDUFA12, DUSP1, SDHB, MT-ND3, MT-ATP8, PSMA7, PSMB6, UBB, PSMB2, PSMB3, NDUFAB1, MT-CYTB, NDUFV2, CALM2, SLC39A3
KEGG_PATHWAY	mmu05014: Amyotrophic lateral sclerosis	ME4	17	4.11E−04	0.039042011	MAP2K3, NDUFB8, NDUFA12, DNAH7A, GPX8, SDHB, MT-ND3, MT-ATP8, PSMA7, PSMB6, PSMB2, PSMB3, NDUFAB1, CASP1, MT-CYTB, NDUFV2, NUP37
KEGG_PATHWAY	mmu05016: Huntington disease	ME4	15	4.94E−04	0.039042011	NDUFB8, NDUFA12, DNAH7A, GPX8, SDHB, MT-ND3, MT-ATP8, PSMA7, PSMB6, PSMB2, PSMB3, NDUFAB1, POLR2D, MT-CYTB, NDUFV2
KEGG_PATHWAY	mmu05022: Pathways of neurodegeneration—multiple diseases	ME4	19	8.04E−04	0.04764275	MAP2K3, NDUFB8, MAP2K2, NDUFA12, DNAH7A, GPX8, SDHB, HSD17B10, MT-ND3, MT-ATP8, PSMA7, PSMB6, UBB, PSMB2, PSMB3, NDUFAB1, MT-CYTB, NDUFV2, CALM2
GOTERM_BP_DIRECT	GO: 0006366 ~ transcription from RNA polymerase II promoter	ME4	15	1.96E−05	0.034801066	GTF2A2, SLC40A1, FOS, SCAF1, FOSL2, SNAPC5, NR4A1, AR, BCL6, POLR2D, FOSB, TAF9B, HMGN3, JUNB, BCL9L
KEGG_PATHWAY	mmu00190: Oxidative phosphorylation	ME7	21	1.34E−13	2.68E−11	MT-ND6, NDUFA13, NDUFA7, NDUFB7, NDUFA6, NDUFA5, NDUFB6, NDUFA4, NDUFA3, COX17, NDUFA1, UQCR11, NDUFC1, UQCR10, ATP5J2, NDUFB4C, UQCRQ, NDUFS6, NDUFS5, ATP5E, NDUFV3
KEGG_PATHWAY	mmu04714: Thermogenesis	ME7	23	6.64E−11	4.85E−09	MT-ND6, NDUFA13, NDUFA7, NDUFB7, NDUFA6, NDUFA5, NDUFB6, NDUFA4, NDUFA3, COX17, NDUFA1, UQCR11, NDUFC1, UQCR10, ATP5J2, NDUFAF8, NDUFB4C, UQCRQ, NDUFS6, NDUFS5, ATP5E, NDUFV3, COX20
KEGG_PATHWAY	mmu04723: Retrograde endocannabinoid signaling	ME7	19	7.28E−11	4.85E−09	MT-ND6, NDUFA13, NDUFA7, NDUFB7, NDUFA6, NDUFA5, SLC32A1, NDUFB6, NDUFA4, NDUFA3, NDUFA1, NDUFC1, GNG11, GNG13, GNGT2, NDUFB4C, NDUFS6, NDUFS5, NDUFV3
KEGG_PATHWAY	mmu05208: Chemical carcinogenesis—reactive oxygen species	ME7	21	1.41E−09	7.05E−08	MT-ND6, NDUFA13, NDUFA7, NDUFB7, NDUFA6, NDUFA5, NDUFB6, NDUFA4, NDUFA3, NDUFA1, CYBA, UQCR11, NDUFC1, UQCR10, VEGFA, NDUFB4C, UQCRQ, NDUFS6, NDUFS5, ATP5E, NDUFV3
KEGG_PATHWAY	mmu05415: Diabetic cardiomyopathy	ME7	20	3.78E−09	1.51E−07	MT-ND6, NDUFA13, NDUFA7, NDUFB7, NDUFA6, NDUFA5, NDUFB6, NDUFA4, NDUFA3, NDUFA1, CYBA, UQCR11, NDUFC1, UQCR10, NDUFB4C, UQCRQ, NDUFS6, NDUFS5, ATP5E, NDUFV3
KEGG_PATHWAY	mmu04932: Non-alcoholic fatty liver disease	ME7	17	1.09E−08	3.64E−07	NDUFA13, NDUFA7, NDUFB7, NDUFA6, NDUFA5, NDUFB6, NDUFA4, NDUFA3, NDUFA1, UQCR11, NDUFC1, UQCR10, NDUFB4C, UQCRQ, NDUFS6, NDUFS5, NDUFV3
KEGG_PATHWAY	mmu05020: Prion disease	ME7	20	1.83E−07	5.22E−06	MT-ND6, NDUFA13, NDUFA7, NDUFB7, NDUFA6, NDUFA5, NDUFB6, NDUFA4, NDUFA3, NDUFA1, CYBA, UQCR11, NDUFC1, UQCR10, NDUFB4C, UQCRQ, NDUFS6, NDUFS5, ATP5E, NDUFV3
KEGG_PATHWAY	mmu05016: Huntington disease	ME7	21	2.57E−07	6.43E−06	MT-ND6, NDUFA13, NDUFA7, NDUFB7, NDUFA6, NDUFA5, NDUFB6, NDUFA4, NDUFA3, NDUFA1, UQCR11, NDUFC1, UQCR10, NDUFB4C, UQCRQ, NDUFS6, NDUFS5, ATP5E, NDUFV3, POLR2J, POLR2L
KEGG_PATHWAY	mmu05012: Parkinson disease	ME7	19	7.01E−07	1.56E−05	MT-ND6, NDUFA13, NDUFA7, NDUFB7, NDUFA6, NDUFA5, NDUFB6, NDUFA4, NDUFA3, NDUFA1, UQCR11, NDUFC1, UQCR10, NDUFB4C, UQCRQ, NDUFS6, NDUFS5, ATP5E, NDUFV3
KEGG_PATHWAY	mmu05014: Amyotrophic lateral sclerosis	ME7	21	5.86E−06	1.17E−04	MT-ND6, RANBP2, NDUFA13, NDUFA7, NDUFB7, NDUFA6, NDUFA5, NDUFB6, NDUFA4, NDUFA3, NDUFA1, UQCR11, NDUFC1, UQCR10, NDUFB4C, UQCRQ, NDUFS6, NDUFS5, ATP5E, NOS1, NDUFV3
KEGG_PATHWAY	mmu05010: Alzheimer disease	ME7	21	1.02E−05	1.86E−04	MT-ND6, NDUFA13, NDUFA7, NDUFB7, NDUFA6, CHRM1, NDUFA5, NDUFB6, NDUFA4, NDUFA3, NDUFA1, UQCR11, NDUFC1, UQCR10, NDUFB4C, UQCRQ, NDUFS6, NDUFS5, ATP5E, NOS1, NDUFV3
KEGG_PATHWAY	mmu05022: Pathways of neurodegeneration—multiple diseases	ME7	22	6.42E−05	0.001069333	MT-ND6, NDUFA13, NDUFA7, NDUFB7, NDUFA6, CHRM1, NDUFA5, NDUFB6, NDUFA4, NDUFA3, NDUFA1, UQCR11, NDUFC1, UQCR10, PDYN, NDUFB4C, UQCRQ, NDUFS6, NDUFS5, ATP5E, NOS1, NDUFV3
GOTERM_CC_DIRECT	GO: 0070469 ~ respiratory chain	ME7	17	1.11E−14	4.46E−12	MT-ND6, NDUFA13, NDUFA7, NDUFB7, NDUFA6, NDUFA5, NDUFB6, NDUFA4, NDUFA3, NDUFA1, UQCR11, NDUFC1, UQCR10, UQCRQ, NDUFS6, NDUFS5, NDUFV3
GOTERM_CC_DIRECT	GO: 0005747 ~ mitochondrial respiratory chain complex I	ME7	15	2.74E−13	5.50E−11	MT-ND6, NDUFA13, NDUFA7, NDUFB7, NDUFA6, NDUFA5, NDUFB6, NDUFA4, NDUFA3, NDUFA1, NDUFC1, NDUFB4C, NDUFS6, NDUFS5, NDUFV3
GOTERM_CC_DIRECT	GO: 0005743 ~ mitochondrial inner membrane	ME7	35	1.18E−10	1.57E−08	MT-ND6, NDUFA13, NDUFB7, NDUFB6, MRPS33, UQCR11, MRPL14, UQCR10, MRPL33, ATP5E, ROMO1, NDUFV3, CHCHD1, DNAJC19, TIMM8B, MRPS28, NDUFA7, NDUFA6, MRPS24, NDUFA5, PET100, UQCC2, NDUFA4, NDUFA3, NDUFA1, MRPS21, NDUFC1, MRPL23, ATP5J2, MRPS18C, TMEM256, UQCRQ, NDUFS6, NDUFS5, COX20
GOTERM_CC_DIRECT	GO: 0005739 ~ mitochondrion	ME7	64	1.31E−06	1.31E−04	MT-ND6, NDUFA13, HJURP, CISD3, MRPL33, GMPPB, NOS1, SERAC1, CHCHD1, DLGAP5, TMEM8B, MRPS28, MRPS24, ATPIF1, MRPS21, CYBA, NDUFC1, ATP5J2, MRPS18C, RPUSD4, QTRT2, NDUFS6, NDUFS5, SDHAF4, FXN, FCOR, NDUFB7, NDUFB6, MRPS33, COX17, CEBPZOS, IBA57, UQCR11, MRPL14, UQCR10, HIGD1B, NT5C, NDUFB4C, ATP5E, MTHFSL, ROMO1, NDUFV3, PPARGC1B, DNAJC19, EEFSEC, RANBP2, TIMM8B, NDUFA7, NDUFA6, PET100, NDUFA5, UQCC2, NDUFA4, NDUFA3, TXNRD1, NDUFA1, MTHFS, MRPL23, LYRM2, NDUFAF8, UQCRQ, FMC1, ATP5MPL, COX20
GOTERM_CC_DIRECT	GO: 0005840 ~ ribosome	ME7	14	6.09E−05	0.004886691	MRPS28, RPS4L, MRPS24, MRPS33, MRPS21, MRPL14, MRPL23, MRPL33, MRPS18C, RPS27, RPS19, RPL35, RPS11, CHCHD1
GOTERM_CC_DIRECT	GO: 0005763 ~ mitochondrial small ribosomal subunit	ME7	6	3.80E−04	0.025389075	MRPS28, MRPS24, MRPS33, MRPS21, CHCHD1, MRPS18C
GOTERM_MF_DIRECT	GO: 0003735 ~ structural constituent of ribosome	ME7	14	2.44E−05	0.012610695	NDUFA7, RPS4L, MRPS24, MRPS21, MRPL14, MRPL23, MRPL33, MRPS18C, RPS27, RPS19, RPS18-PS5, RPS27RT, RPL35, RPS11
GOTERM_BP_DIRECT	GO: 0042776 ~ mitochondrial ATP synthesis coupled proton transport	ME7	15	2.78E−12	4.88E−09	MT-ND6, NDUFA13, NDUFA7, NDUFB7, NDUFA6, NDUFA5, NDUFB6, NDUFA3, NDUFA1, NDUFC1, ATP5J2, NDUFS6, NDUFS5, ATP5E, NDUFV3
GOTERM_BP_DIRECT	GO: 0009060 ~ aerobic respiration	ME7	15	1.88E−11	1.64E−08	MT-ND6, NDUFA13, NDUFA7, NDUFB7, NDUFA6, NDUFA5, NDUFB6, NDUFA3, NDUFA1, NDUFC1, UQCR10, NDUFS6, NDUFS5, NDUFV3, FXN
GOTERM_BP_DIRECT	GO: 0032981 ~ mitochondrial respiratory chain complex I assembly	ME7	13	4.72E−10	2.76E−07	MT-ND6, NDUFA13, NDUFB7, NDUFA6, NDUFA5, NDUFB6, NDUFA3, NDUFA1, NDUFC1, NDUFAF8, NDUFB4C, NDUFS6, NDUFS5
GOTERM_BP_DIRECT	GO: 0032543 ~ mitochondrial translation	ME7	10	3.41E−05	0.014945752	MRPS28, NDUFA7, MRPS24, MRPS33, MRPS21, MRPL14, MRPL23, CHCHD1, MRPL33, MRPS18C
KEGG_PATHWAY	mmu03010: Ribosome	ME8	23	3.38E−26	1.93E−24	RPL30, RPL10, RPLP1, RPL11, RPL22, RPSA, N-R5S121, N-R5S143, N-R5S113, N-R5S111, N-R5S144, N-R5S128, RPS3A1, RPL27A, GM25018, N-R5S138, RPS20, RPS2, RPS27A, RPL18, RPS10, RPS13, RPS23
KEGG_PATHWAY	mmu05171: Coronavirus disease—COVID-19	ME8	15	1.09E−11	3.10E−10	RPL30, RPL10, RPLP1, RPL11, RPL22, RPSA, RPS3A1, RPL27A, RPS20, RPS2, RPS27A, RPL18, RPS10, RPS13, RPS23
KEGG_PATHWAY	mmu03008: Ribosome biogenesis in eukaryotes	ME8	8	1.94E−06	3.68E−05	N-R5S113, N-R5S111, N-R5S144, N-R5S128, N-R5S138, GM25018, N-R5S121, N-R5S143
GOTERM_CC_DIRECT	GO: 0022626 ~ cytosolic ribosome	ME8	15	1.43E−20	2.26E−18	RPL30, RPL10, RPLP1, RPL11, RPL22, RPSA, RPS3A1, RPL27A, RPS20, RPS2, RPS27A, RPL18, RPS10, RPS13, RPS23
GOTERM_CC_DIRECT	GO: 0005840 ~ ribosome	ME8	15	4.60E−15	3.63E−13	RPL30, RPL10, RPLP1, RPL11, RPL22, RPSA, RPS3A1, RPL27A, RPS20, RPS2, RPS27A, RPL18, RPS10, RPS13, RPS23
GOTERM_CC_DIRECT	GO: 0022627 ~ cytosolic small ribosomal subunit	ME8	9	4.26E−12	2.25E−10	RPS3A1, GM49804, RPSA, RPS20, RPS2, RPS27A, RPS10, RPS13, RPS23
GOTERM_CC_DIRECT	GO: 0045202 ~ synapse	ME8	21	1.00E−10	3.95E−09	RPL30, PTPRN2, RPL10, RPLP1, RPL11, RPL22, HSPB1, RPSA, RPS3A1, RPL27A, EEF1A2, RPS20, RPS2, DLGAP3, RPS27A, RPL18, RPS10, RPS13, BCAS1, YWHAH, RPS23
GOTERM_CC_DIRECT	GO: 0098794 ~ postsynapse	ME8	13	3.10E−10	9.81E−09	RPL30, RPL10, RPLP1, RPL22, RPSA, FXR2, RPS3A1, RPL27A, RPS20, RPS2, RPS27A, RPS10, RPS23
GOTERM_CC_DIRECT	GO: 0022625 ~ cytosolic large ribosomal subunit	ME8	8	4.12E−09	1.08E−07	RPL30, RPL10, RPL27A, RPLP1, RPL11, RPL22, RPL18, RPL9-PS6
GOTERM_CC_DIRECT	GO: 0015935 ~ small ribosomal subunit	ME8	5	1.78E−06	4.02E−05	RPS3A1, RPSA, RPS20, RPS2, RPS23
GOTERM_CC_DIRECT	GO: 0005829 ~ cytosol	ME8	31	2.54E−05	5.01E−04	RPL30, RPL10, RPLP1, URM1, RPL11, CXXC1, SEC14L2, RGS5, FXR2, KLC2, COTL1, RPS2, RPS27A, RPL18, RPS10, RPS13, WDTC1, BCAS1, YWHAH, CAP1, DAPK3, RPL22, IRGM1, RPSA, MT2, MED27, FMNL1, RPS3A1, RPL27A, RPS20, RPS23
GOTERM_CC_DIRECT	GO: 0098793 ~ presynapse	ME8	8	5.05E−05	8.70E−04	FXR2, PTPRN2, RPL10, RPL27A, RPL22, RPS27A, RPS10, YWHAH
GOTERM_CC_DIRECT	GO: 0005737 ~ cytoplasm	ME8	43	5.51E−05	8.70E−04	CEP57, MACF1, RPL30, RPL10, RPLP1, URM1, RPL11, HSPB1, HSD17B11, PPP1R18, SEC14L2, RGS5, FXR2, KLC2, CSRP1, NPHP1, COTL1, RPS2, RPS27A, RPL18, RPS10, TSPOAP1, RPS13, WDTC1, BCAS1, YWHAH, CAP1, PTPRN2, TAF10, DAPK3, RPL22, HAUS3, RPSA, MT2, TPPP3, FMNL1, RPS3A1, RPL27A, ID1, EEF1A2, RPS20, SFI1, RPS23
GOTERM_CC_DIRECT	GO: 0042788 ~ polysomal ribosome	ME8	4	2.33E−04	0.003340004	RPL30, RPL11, RPL18, RPS23
GOTERM_MF_DIRECT	GO: 0003735 ~ structural constituent of ribosome	ME8	18	2.91E−19	4.63E−17	RPL30, RPL10, RPLP1, GM49804, RPL11, RPL22, RPSA, RPL9-PS6, RPS3A1, RPL27A, RPS20, RPS2, GM49711, RPS27A, RPL18, RPS10, RPS13, RPS23
GOTERM_BP_DIRECT	GO: 0002181 ~ cytoplasmic translation	ME8	15	2.79E−20	1.21E−17	RPL30, RPLP1, RPL11, RPL22, RPSA, RPL9-PS6, RPS3A1, RPL27A, RPS20, RPS2, RPS27A, RPL18, RPS10, RPS13, RPS23
GOTERM_BP_DIRECT	GO: 0006412 ~ translation	ME8	15	1.14E−12	2.48E−10	RPL10, RPL11, RPL22, RPSA, LARS2, RPS3A1, RPL27A, EEF1A2, RPS20, RPS2, GM49711, RPS27A, RPL18, RPS13, RPS23

*Corrected by the Benjamini–Hochberg method

The analysis was performed by DAVID software

**Table 2:** GO (biological process) over representation analysis of ME3 module and list of involved genes in wild-type mice and IL-1b KO mice exposed to acrylamide at 0, 12.5, 25 mg/kg for 28 days by oral gavage

Gene name	Regulation	*q* value	Fold change	Wild-type (mg/kg) (mean ± SD)			IL-1b KO (mg/kg) (mean ± SD)			GO/KEGG term
				0	12.5	25	0	12.5	25	
SNORC (*Secondary Ossification Center Associated Regulator of Chondrocyte Maturation)*	Upregulated	2.05E−07	4.608	2.93 ± 0.974	2.63 ± 2.45	4.41 ± 2.19	8.51 ± 4.0	13 ± 3.8	12.52 ± 4.94	GO: 0005576 ~ extracellular region
PFN1 (Profilin 1)	Upregulated	2.81E−17	1.788	384.52 ± 46.48	486.09 ± 43.51	442.1 ± 41.3	610.58 ± 47.46	687.7 ± 48.8	683 ± 81.33	
CRHBP (Corticotropin-releasing hormone binding protein)	Upregulated	6.21E−09	1.572	203.34 ± 21.6	219.6 ± 15.6	246.9 ± 22.2	301.7 ± 21.3	319.6 ± 37.0	276.5 ± 18.14	
PAMR1 (Peptidase domain containing associated with muscle regeneration)	Upregulated	0.002867	1.539	74.4 ± 12.9	88.2 ± 6.97	93 ± 8.55	100.8 ± 14.1	114.6 ± 11.7	104.18 ± 21.96	
LY86 (Lymphocyte antigen 86)	Downregulated	5.84E−27	0.435	95 ± 17.4	110.4 ± 20.3	120.4 ± 13.1	43.74 ± 8.0	41.4 ± 7.7	50.1 ± 7	
MYOC (Myocilin)	Downregulated	5.77E−08	0.546	36.13 ± 6.4	52.45 ± 12.2	45.82 ± 8.41	25.5 ± 12.88	19.73 ± 7.54	24.84 ± 15.19	
NMI (N-myc-interactor	Downregulated	0.000248	0.559	36.05 ± 8.43	37.41 ± 5.39	42.85 ± 6.73	26.93 ± 6.38	27.57 ± 16.61	20.15 ± 5.64	

DAVID software was used for analysis. Data of normalized values for gene expression are mean ± SD, *n* = 6. All *P* value for gene expression were adjusted using Benjamini–Hochberg method and expressed as *q* values. Fold change represents the ratio of the mean value of the group to the mean of the 0 mg/kg wild-type group when the absolute value of logarithm of the ratio is the maximum

**Fig. 2:**
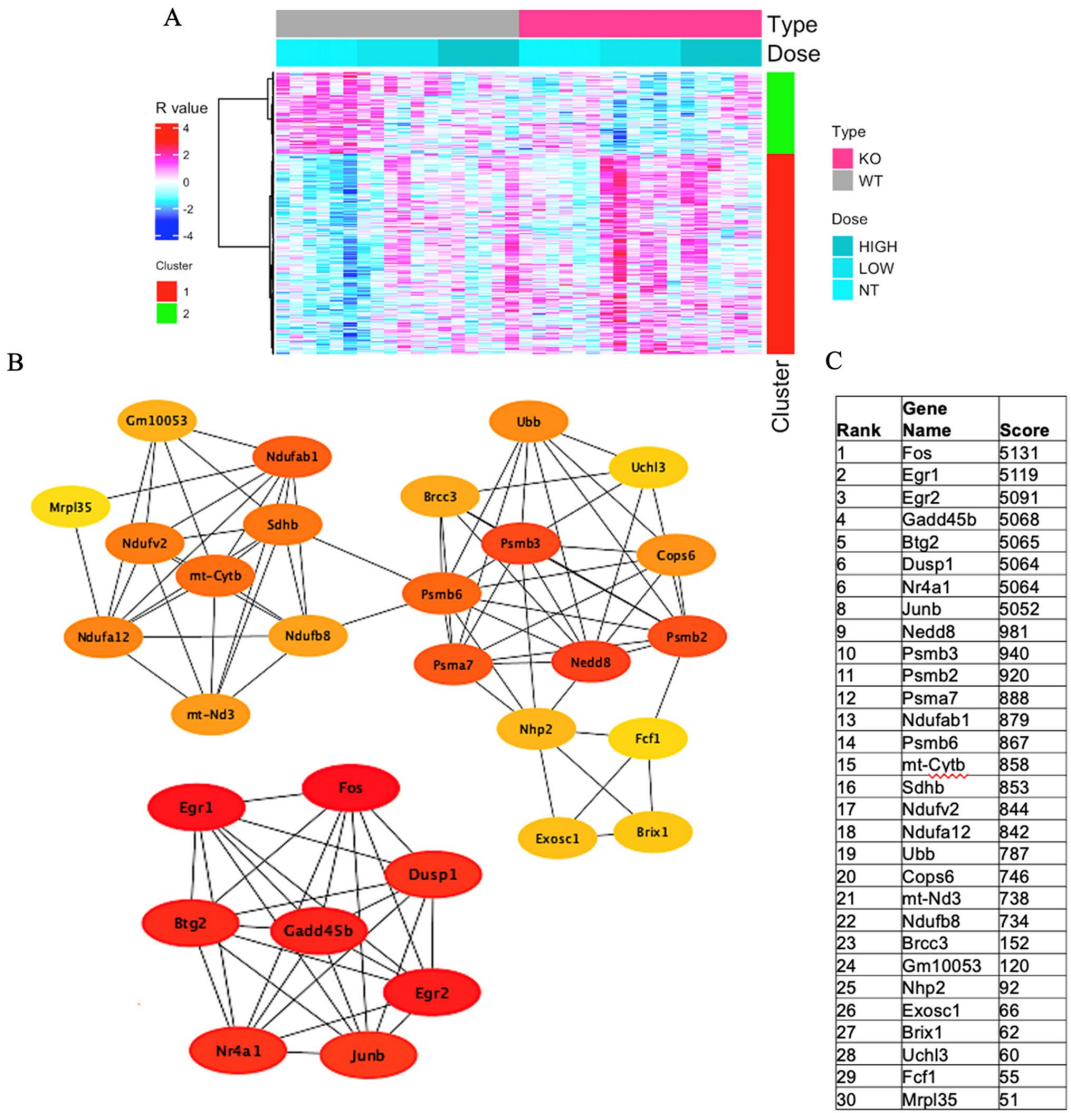
**A** Heatmap of module eigengene 4 (ME4, 417 genes) of the cerebral cortex of acrylamide-exposed WT and IL-1ß KO mice. Columns: exposure groups (0, 12.5, 25 mg/kg). Red cluster (1): upregulated genes, green cluster *(2):* downregulated genes. **B** Protein network analysis of ME4 module using STRING software. **C** Table shows the scores of the top proteins in the ME4 arranged from the highest score (top) to the lowest score (bottom) (color figure online)

Similar analysis in module eigengene 4 (ME4) (Fig. 2A), which provides assessment of ACR effect on gene expression both in the wild-type and *IL-*1β KO, showed overexpression of 417 genes. The analysis showed that the KEGG mmu05012: Parkinson disease (*q* = 0.007), KEGG mmu05014: amyotrophic lateral sclerosis (*q* = 0.039), KEGG mmu05016: Huntington disease (*q* = 0.039), KEGG mmu05022: Pathways of neurodegeneration—multiple diseases (*q* = 0.047) and GO-BP: 0006366 ~ transcription from RNA polymerase II promoter (*q* = 0.034) were significantly expressed in this module (Table 1). The following genes were upregulated significantly: NDUFB8 (*q* = 3.20E−06), MT-ATP8 (*q* = 6.33E−10), PSMB6 (*q* = 4.48E−07), NDUFA12 (8.91E−06), DNAH7A (0.072927), PSMB3 (*q* = 1.76E−07), and MT-CYTB (*q* = 8.06E−06), while the following genes were significantly downregulated: DUSP1 (*q* = 0.0002670), MT-ND3 (*q* = 0.013479), and SLC39A3 (*q* = 0.044957) (Table 3).

**Table 3:** GO (biological process) and KEGG pathway over representation analysis of ME4 module and list of involved genes in acrylamide-exposed wild-type mice and IL-1b KO mice groups at 0, 12.5, 25 mg/kg for 28 days by oral gavage

Gene name	Regulation	*q* value	Fold change	Wild-type (mg/kg) (mean ± SD)			IL-1b KO (mg/kg) (mean ± SD)			GO/KEGG term
				0	12.5	25	0	12.5	25	
NDUFB8	Upregulated	3.20E−06	1.577	3394 ± 467	4460 ± 591	4465 ± 468	4161 ± 316	5355 ± 478	4890 ± 1132	KEGG-mmu05012: PD, KEGG-mmu05014: ALS, KEGG-mmu05016: Huntington disease, KEGGmmu05022: Pathways of neurodegeneration—multiple diseases
MT-ATP8	Upregulated	6.33E−10	1.757	62,983 ± 13,582	755,528 ± 8682	80,676 ± 13,604	84,387 ± 9436	110,711 ± 14,621	105,250 ± 16,832	KEGG-mmu05012: PD, KEGG-mmu05014: ALS, KEGG-mmu05016: Huntington disease, KEGGmmu05022: Pathways of neurodegeneration—multiple diseases
PSMB6	Upregulated	4.48E−07	1.588	1639 ± 147	2220 ± 281	2230 ± 173	1980 ± 148	2603 ± 230	2340 ± 518	KEGG-mmu05012: PD, KEGG-mmu05014: ALS, KEGG-mmu05016: Huntington disease, KEGG-mmu05022: Pathways of neurodegeneration—multiple diseases
NDUFA12	Upregulated	8.91E−06	1.558	2240 ± 283	2764 ± 330	2805 ± 300	2706 ± 186	3491 ± 493	3141 ± 740	KEGG-mmu05012: PD, KEGG-mmu05014: ALS
DNAH7A	Upregulated	0.072927	1.571	35.30 ± 6.7	49 ± 10	46 ± 6.9	50 ± 9.3	56 ± 6.4	53 ± 13	KEGG-mmu05012: PD, KEGG-mmu05014: ALS
PSMB3	Upregulated	1.76E−07	1.559	736 ± 62.8	1009 ± 98	974 ± 107	869 ± 51	1148 ± 177	1081 ± 162	KEGG-mmu05014: ALS, KEGG-mmu05016: Huntington disease, KEGGmmu05022: Pathways of neurodegeneration—multiple diseases
MT-CYTB	Upregulated	8.06E−06	1.526	36,367 ± 5894	40,408 ± 2631	443,691 ± 10,266	48,664 ± 4571	555,194 ± 6567	51,310 ± 7948	KEGG-mmu05012: PD
DUSP1	Downregulated	0.000267	0.518	110 ± 29	106 ± 31	76 ± 30	57 ± 22	80 ± 24	80 ± 36	KEGG-mmu05012: PD
MT-ND3	Downregulated	0.013479	0.699	2942 ± 300	2681 ± 501	2569 ± 620	2836 ± 369	2059 ± 675	26,371 ± 508	KEGG-mmu05012: PD, KEGG-mmu05014: ALS, KEGG-mmu05016: Huntington disease, KEGG-mmu05022: Pathways of neurodegeneration—multiple diseases
SLC39A3	Downregulated	0.044957	0.709	104 ± 18	89 ± 17	74 ± 14	76 ± 12	81 ± 8	83 ± 5.8	KEGG-mmu05012: PD

DAVID software was used for the analysis. Data of normalized values for gene expression are mean ± SD, *n* = 6. All *P* value for the gene expression were adjusted using Benjamini–Hochberg method and expressed as q values. Fold change represents the ratio of the mean value of the group to the mean of the 0 mg/kg wild-type when the absolute value of the logarithm of the ratio is the maximum

*PD* Parkinson disease, *ALS* amyotrophic lateral sclerosis, *NDUFB8* NADH: ubiquinone oxidoreductase subunit B8, *MT-ATP8* mitochondrially encoded ATP synthase membrane subunit 8, *PSMB6* proteasome 20S subunit beta 6, *NDUFA12* NADH: ubiquinone oxidoreductase subunit A12, *DNAH7A* dynein, axonemal, heavy chain 7A, *PSMB3* proteasome subunit beta type-3, *MT-CYTB* mitochondrially encoded cytochrome B, *DUSP1* dual specificity phosphatase 1, *MT-ND3* mitochondrially encoded NADH: ubiquinone oxidoreductase core subunit 3, *SLC39A3* solute carrier family 39 member 3

Protein network analysis of ME4 of genes with q value < 0.05 showed involvement of different proteins that are highly expressed and involved in cell proliferation, such as Fos and Btg2, Egr1 and Egr2, and Nr4a proteins involved in memory and learning. Other groups of proteins also showed high scores, including proteasomal protein Psmb2, Psmb3, Psmb6, and Psma7. Mitochondrial proteins Nudfab1, Nudfa12, Nudfv2, mt-Cytb, and Sdhb also showed interaction, indicating the involvement of oxidative stress (Fig. 2B, C).

**Fig. 3:**
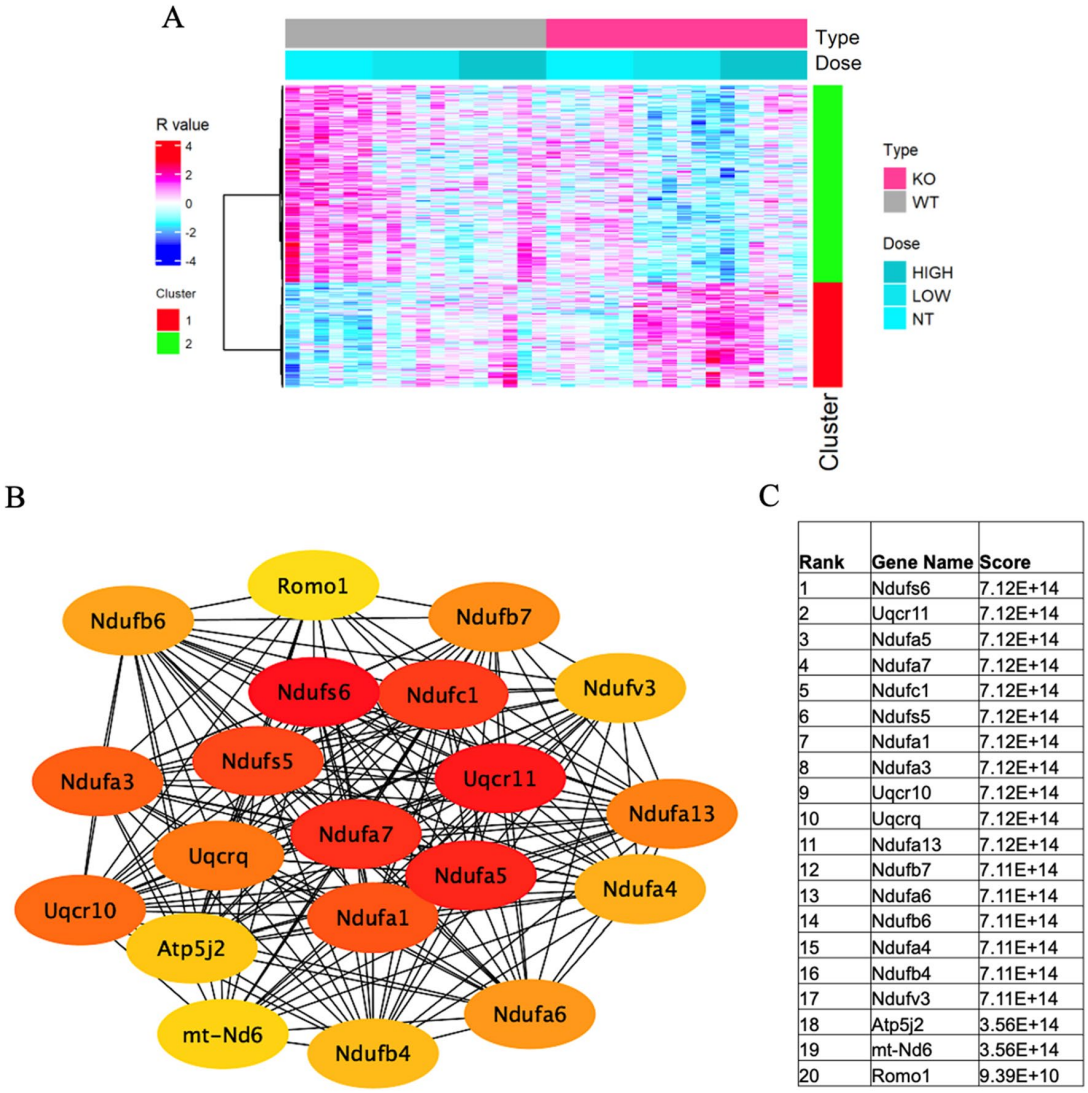
**A** Heatmap of module eigengene 4 (ME7, 578 genes) of the cerebral cortex of acrylamide-exposed WT and *IL-1ß* KO mice. Columns: exposure groups (0, 12.5, 25 mg/kg). Red cluster (1): upregulated genes, green cluster (2): downregulated genes. **B** Protein network analysis of ME7 module using STRING software. **C** Table shows the scores of top proteins in the ME7 arranged from the highest score (top) to the lowest score (bottom) (color figure online)

Similar analysis of module eigengene 7 (ME7) (Fig. 3A) showed high expression of 578 genes. Specifically, the analysis identified overexpression of KEGG mmu00190: Oxidative phosphorylation (*q* = 2.68E−11), KEGG mmu04714: Thermogenesis (*q* = 4.85E−09), KEGG mmu04723: Retrograde endocannabinoid signaling (*q* = 4.85E−09), KEGG mmu05208: Chemical carcinogenesis-reactive oxygen species (*q* = 7.05E−08), KEGG mmu05415: Diabetic cardiomyopathy (*q* = 1.51E−07), KEGG mmu05020: Prion disease (*q* = 5.22E−06), KEGG mmu05016: Huntington disease (*q* = 6.43E−06), KEGG mmu05012: Parkinson disease (*q* = 1.56E−05), KEGG mmu05014: Amyotrophic lateral sclerosis (*q* = 1.17E−04), KEGG mmu05010: Alzheimer disease (*q* = 1.86E−04), KEGG mmu05022: Pathways of neurodegeneration-multiple diseases (*q* = 0.001), KEGG mmu04932: Non-alcoholic fatty liver disease (*q* = 3.64E−07), GO-CC: 0070469-respiratory chain (*q* = 4.46E−12), GO-CC: 0005747-mitochondrial respiratory chain complex I (*q* = 5.50E−11), GO-CC O: 0005743-mitochondrial inner membrane (*q* = 1.57E−08), GO-CC: 0005739-mitochondrion (*q* = 1.31E−04), GO-CC: 0005840-ribosome (*q* = 0.004), GO-CC: 0005763-mitochondrial small ribosomal subunit (*q* = 0.025), GO-MF: 0003735-structural constituent of ribosome (*q* = 0.0126), GO-BP: 0042776-mitochondrial ATP synthesis coupled proton transport (*q* = 4.88E−09), GO-BP: 0009060-aerobic respiration (*q* = 1.64E−08), GO-BP: 0032981-mitochondrial respiratory chain complex I assembly (*q* = 2.76E−07), and GO-BP: 0032543-mitochondrial translation (*q* = 0.014) (Table 1).

The following genes showed significant upregulation in the ME7 module: CEBPZOS (*q* = 1.10E−22), NDUFS6 (*q* = 1.48E−14), SDHAF4 (*q* = 6.04E−10), NDUFA13 (*q* = 5.93E−11), NDUFB7 (*q* = 2.27E−10), NDUFA3 (*q* = 1.32E−09), MRPL14 (*q* = 5.46E−10), CYBA (*q* = 0.001343), MRPL33 (*q* = 2.10E−06), RPL35 (*q* = 0.000127), MRPS33 (*q* = 1.63E−05), MRPS21 (*q* = 2.32E−06), NDUFS5 (*q* = 3.84E−06), and MRPS28 (3.84E−05). On the other hand, significant downregulation of RPS4L (*q* = 4.88E−05) and MT-ND6 (*q* = 5.76E−15) genes was noted (Table 4). Protein network analysis of the ME7 module showed the association of multiple mitochondrial proteins, including Ndufs6, uqcr11, Ndufa5, Ndufa7, Ndufc1, Ndufs5, Ndufa1, Ndufa3, Uqrc10, Uqcrq, Ndufa13, Ndufb7, Ndufa6, Ndufb6, Ndufa4, and Ndufv3, with high scores, indicating possible involvement of these proteins in ACR-induced oxidative stress and subsequent neurotoxicity (Fig. 3B, C).

**Table 4:** GO (biological process) and KEGG pathway over representation analysis of ME7 module and list of involved genes in wild-type and IL-1b KO mice exposed to acrylamide at 0, 12.5, 25 mg/kg for 28 days by oral gavage

Gene	Regulation	*q* value	Fold change	Wild type (mg/kg) (mean ± SD)			IL-1b KO (mg/kg) (mean ± SD)			GO/KEGG term
				0	12.5	25	0	12.5	25	
CEBPZOS	Upregulated	1.10E−22	2.409	666 ± 78	896.93 ± 116	892 ± 89	787 ± 51	1606 ± 199	1291 ± 471	GO-CC: 0005739 ~ mitochondrion
NDUFS6	Upregulated	1.48E E−14	1.874	14,924 ± 131	1923 ± 157	1936 ± 326	2134 ± 111	2797 ± 334	2591 ± 490	KEGG-mmu00190: Oxidative phosphorylation, mmu04714: Thermogenesis, mmu04723: Retrograde endocannabinoid signaling, mmu05208: Chemical carcinogenesis-ROS, mmu05415: Diabetic cardiomyopathy, mmu05020: Prion disease, mmu05016: Huntington disease, mmu05012: PD, mmu05014: ALS, mmu05010: AD, mmu05022: Pathways of neurodegeneration—multiple diseases, mmu04932: Non-alcoholic fatty liver disease, GO-CC GO: 0070469 ~ respiratory chain, GO: 0005747 ~ mitochondrial respiratory chain complex I,GO: 0005743 ~ mitochondrial inner membrane, GO: 0005739 ~ mitochondrion, GO-BP: 0042776 ~ mitochondrial ATP synthesis coupled proton transport, GO-BP: 0009060 ~ aerobic respiration, GO-BP: 0032981 ~ mitochondrial respiratory chain complex I assembly
SDHAF4	Upregulated	6.04E E−10	1.768	927 ± 101	1354 ± 203	1373 ± 217	1173 ± 91	1639 ± 136	1469 ± 362	GO-CC: 0005739 ~ mitochondrion
NDUFA13	Upregulated	5.93E E−11	1.747	2873 ± 273	3651 ± 480	3699 ± 426	3552 ± 328	5019 ± 591	4536 ± 877	KEGG-mmu00190: Oxidative phosphorylation, mmu04714: Thermogenesis, mmu04723: Retrograde endocannabinoid signaling, mmu05208: Chemical carcinogenesis—ROS, mmu05415: Diabetic cardiomyopathy, mmu05020: Prion disease, mmu05016: Huntington disease, mmu05012: PD, mmu05014: ALS, mmu05010: AD, mmu05022: Pathways of neurodegeneration—multiple diseases, mmu04932: Non-alcoholic fatty liver disease, GO-CC GO: 0070469 ~ respiratory chain, GO: 0005747 ~ mitochondrial respiratory chain complex I,GO: 0005743 ~ mitochondrial inner membrane, GO: 0005739 ~ mitochondrion, GO-BP: 0042776 ~ mitochondrial ATP synthesis coupled proton transport, GO-BP: 0009060 ~ aerobic respiration, GO-BP: 0032981 ~ mitochondrial respiratory chain complex I assembly
NDUFB7	Upregulated	2.27E E−10	1.745	1376 ± 149	18,855 ± 253	1816 ± 156	1756 ± 76	2403 ± 256	2073 ± 322	KEGG-mmu00190: Oxidative phosphorylation, mmu04714: Thermogenesis, mmu04723: Retrograde endocannabinoid signaling, mmu05208: Chemical carcinogenesis—ROS, mmu05415: Diabetic cardiomyopathy, mmu05020: Prion disease, mmu05016: Huntington disease,mmu05012: PD, mmu05014: ALS, mmu05010: AD, mmu05022: Pathways of neurodegeneration—multiple diseases, mmu04932: Non-alcoholic fatty liver disease, GO-CC GO: 0070469-respiratory chain, GO: 0005747-mitochondrial respiratory chain complex I,GO: 0005743-mitochondrial inner membrane, GO: 0005739-mitochondrion, GO-BP: 0042776-mitochondrial ATP synthesis coupled proton transport, GO-BP: 0009060 ~ aerobic respiration, GO-BP: 0032981 ~ mitochondrial respiratory chain complex I assembly
NDUFA3	Upregulated	1.32E E−09	1.740	2280 ± 171	2926 ± 255	2988 ± 568	2664 ± 174	3968 ± 880	3439 ± 678	KEGG-mmu00190: Oxidative phosphorylation, mmu04714: Thermogenesis, mmu04723: Retrograde endocannabinoid signaling, mmu05208: Chemical carcinogenesis—ROS, mmu05415: Diabetic cardiomyopathy, mmu05020: Prion disease, mmu05016: Huntington disease,mmu05012: PD,mmu05014: ALS,mmu05010: AD, mmu05022: Pathways of neurodegeneration—multiple diseases, mmu04932: Non-alcoholic fatty liver disease, GO-CC GO: 0070469-respiratory chain, GO: 0005747-mitochondrial respiratory chain complex I,GO: 0005743 ~ mitochondrial inner membrane, GO: 0005739 ~ mitochondrion, GO-BP: 0042776-mitochondrial ATP synthesis coupled proton transport, GO-BP: 0009060-aerobic respiration, GO-BP: 0032981-mitochondrial respiratory chain complex I assembly
MRPL14	Upregulated	5.46E E−10	1.7126	503 ± 40	732 ± 94	705 ± 86	632 ± 52	862 ± 101	792 ± 142	GO-CC: 0005840-ribosome, GO-MF: 0003735-structural constituent of ribosome, GO-BP: 0032543-mitochondrial translation
CYBA	Upregulated	0.001343	1.693	43 ± 5.6	50.9 ± 10	50.7 ± 4.7	49.3 ± 9.1	59.7 ± 10.3	72.2 ± 12.8	KEGG-mmu05208: Chemical carcinogenesis—ROS, mmu05415: Diabetic cardiomyopathy, mmu05020: Prion disease
MRPL33	Upregulated	2.10E E−06	1.623	643 ± 71	808 ± 134	858 ± 130	766 ± 90	1043 ± 175	949 ± 262	GO-CC: 0005840 ~ ribosome, GO-MF: 0003735 ~ structural constituent of ribosome, GO-BP: 0032543 ~ mitochondrial translation
RPL35	Upregulated	0.000127	1.604	409 ± 41.9	509 ± 76	477 ± 51	473 ± 32	607 ± 82	539 ± 84	GO-CC: 0005840 ~ ribosome
MRPS33	Upregulated	1.63E E−05	1.537	2104 ± 173	2786 ± 408	2725 ± 282	2533 ± 233	3235 ± 314	2958 ± 735	GO-CC: 0005840 ~ ribosome, GO: 0005763 ~ mitochondrial small ribosomal subunit, GO-BP: 0032543 ~ mitochondrial translation
MRPS21	Upregulated	2.32E E−06	1.537	1034 ± 93	1394 ± 185	1387 ± 224	1206 ± 25	1592 ± 228	1459 ± 254	GO-CC: 0005840 ~ ribosome, GO: 0005763 ~ mitochondrial small ribosomal subunit, GO-BP: 0032543 ~ mitochondrial translation
NDUFS5	Upregulated	3.84E E−06	1.552	3073 ± 282	4023 ± 472	3960 ± 741	3566 ± 325	4772 ± 654	4372 ± 833	GO-BP: 0042776 ~ mitochondrial ATP synthesis coupled proton transport, GO: 0009060 ~ aerobic respiration, GO: 0032981 ~ mitochondrial respiratory chain complex I assembly
MRPS28	Upregulated	3.84E E−05	1.509	292 ± 32	376 ± 47	370 ± 42	352 ± 33	440 ± 50	420 ± 82	GO-CC: 0005840 ~ ribosome, GO-CC: 0005739 ~ mitochondrion, GO: 0005763 ~ mitochondrial small ribosomal subunit
RPS4L	Down regulated	4.88E E−05	0.695	147 ± 26	168 ± 29	137 ± 27	124 ± 14	102 ± 15	119 ± 25	GO-CC: 0005840 ~ ribosome
MT-ND6	Down regulated	5.76E E−15	0.509	1338 ± 126	1086 ± 175	978 ± 146	1182 ± 1493	682 ± 100	821 ± 164	KEGG-mmu00190: Oxidative phosphorylation, mmu04714: Thermogenesis, mmu04723: Retrograde endocannabinoid signaling, mmu05208: Chemical carcinogenesis—ROS, mmu05415: Diabetic cardiomyopathy, mmu05020: Prion disease, mmu05016: Huntington disease, mmu05012: PD, mmu05014: ALS,mmu05010: AD, mmu05022: Pathways of neurodegeneration—multiple diseases, mmu04932: Non-alcoholic fatty liver disease, GO-CC GO: 0070469 ~ respiratory chain, GO: 0005747 ~ mitochondrial respiratory chain complex I, GO: 0005743 ~ mitochondrial inner membrane, GO: 0005739 ~ mitochondrion, GO-BP: 0042776 ~ mitochondrial ATP synthesis coupled proton transport, GO-BP: 0009060 ~ aerobic respiration, GO-BP: 0032981 ~ mitochondrial respiratory chain complex I assembly

DAVID software was used for analysis. Data of normalized values for gene expression are mean ± SD, *n* = 6. Fold change represents the ratio of the group mean relative to the mean value of the 0 mg/kg wild-type group when the absolute value of logarithm of the ratio is the maximum. All *P* values for gene expression were adjusted using Benjamini–Hochberg method and expressed as q values

*PD* Parkinson’s disease, *ALS* amyotrophic lateral sclerosis, *AD* Alzheimer disease, *ROS* reactive oxygen species CEBPZOS, *CEBPZ Opposite Strand*, *NDUFS6* NADH: ubiquinone oxidoreductase subunit S6, *SDHAF4* succinate dehydrogenase complex assembly factor 4, *NDUFA13* NADH: ubiquinone oxidoreductase subunit A13; NDUFB7, NADH: ubiquinone oxidoreductase subunit b7, *NDUFA3* NADH: ubiquinone oxidoreductase subunit A3, *MRPL14* mitochondrial ribosomal protein L14, *CYBA* cytochrome B-245 alpha chain, *MRPL33* mitochondrial ribosomal protein L33, *RPL35* ribosomal protein L35, *MRPS33* mitochondrial ribosomal protein S33, *MRPS21* mitochondrial ribosomal protein S2, *NDUFS5* NADH: ubiquinone oxidoreductase subunit S5, *MRPS28* mitochondrial ribosomal protein S28, *RPS4L* ribosomal protein S4-like, *MT-ND6* mitochondrially encoded NADH: ubiquinone oxidoreductase core subunit 6

**Fig. 4:**
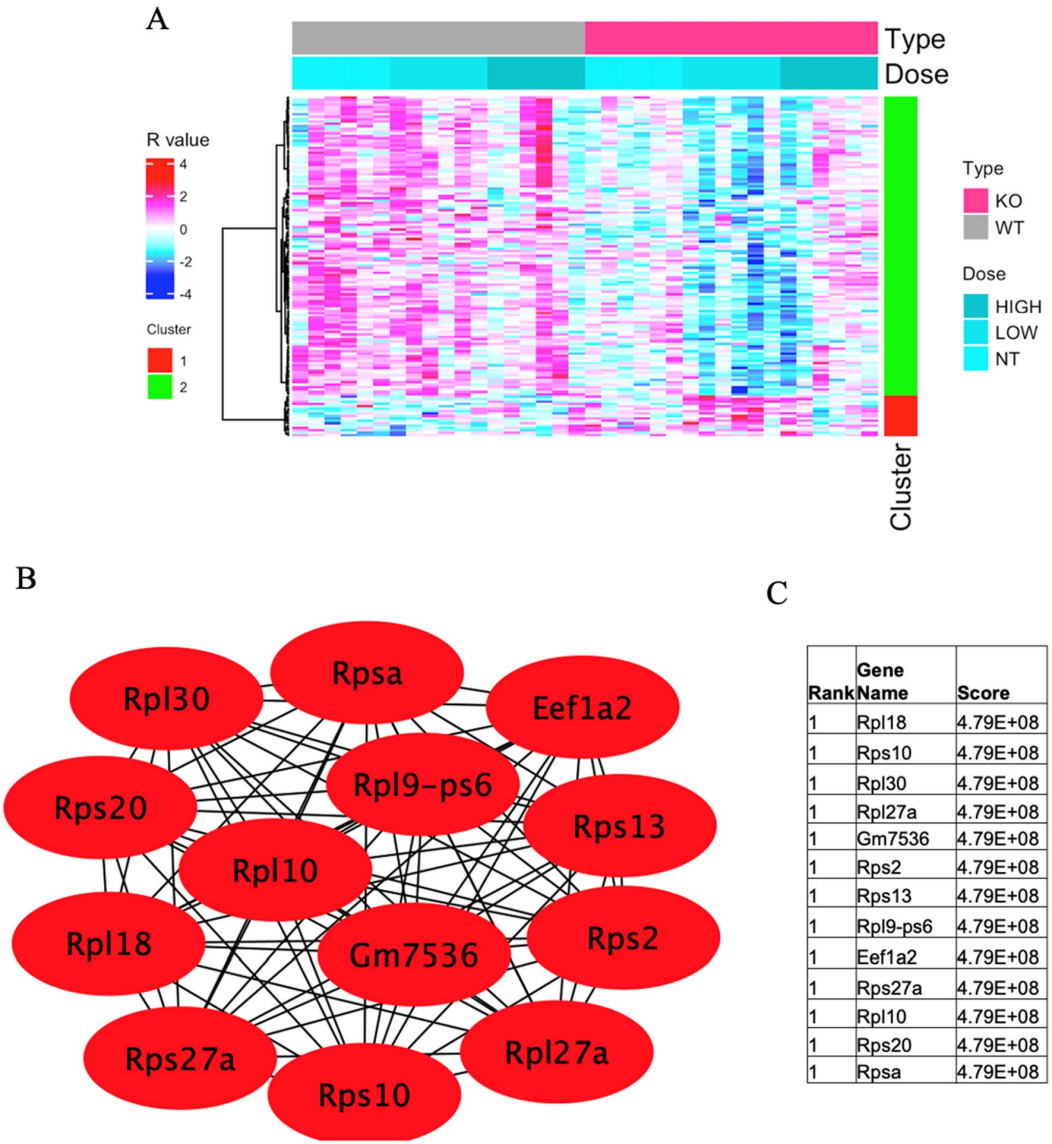
**A** Heatmap of module eigengene 4 (ME8, 142 genes) of the cerebral cortex of acrylamide-exposed WT and *IL-1ß* KO mice. Columns: exposure groups (0, 12.5, 25 mg/kg). Red cluster (1): upregulated genes, green cluster (2): downregulated genes. **B** Protein network analysis of ME8 module using STRING software. **C** Table shows the scores of the top proteins in ME8 arranged from the highest score (top) to the lowest score (bottom) (color figure online)

In module eigengene 8 (ME8) (Fig. 4A), increased expression of 142 genes was noted in different GO and KEGG by David software. The analysis showed various GO and KEGG pathways with significant expression, including KEGG mmu03010: Ribosome (*q* = 1.93E−24), KEGG mmu05171: Coronavirus disease—COVID-19 (*q* = 3.10E−10), KEGG mmu03008: Ribosome biogenesis in eukaryotes (*q* = 3.68E−05), GO-CC: 0022626-cytosolic ribosome (*q* = 2.26E−18), GO-CC: 0005840-ribosome (*q* = 3.63E−13), GO-CC: 0022627-cytosolic small ribosomal subunit (*q* = 2.25E−10), GO-CC: 0045202-synapse (*q* = 3.95E−09), GO-CC: 0098794-postsynapse (*q* = 9.81E−09), GO-CC: 0022625-cytosolic large ribosomal subunit (*q* = 1.08E−07), GO-CC: 0015935-small ribosomal subunit (*q* = 4.02E−05), GO-CC: 0005829-cytosol (*q* = 5.01E−04), GO-CC: 0098793-presynapse (*q* = 8.70E−04), GO-CC: 0005737-cytoplasm (*q* = 8.70E−04), GO-CC: 0042788-polysomal ribosome (*q* = 0.003), GO-MF: 0003735- ~ structural constituent of ribosome (*q* = 4.63E−17), GO-BP: 0002181-cytoplasmic translation (*q* = 1.21E−17), and GO-BP: 0006412-translation (*q* = 2.48E−10) (Table 1).

The following genes were upregulated in the ME8 module: MACF1 (*q* = 1.27E−20), YWHAH (*q* = 5.08E−05), MT2 (*q* = 0.00586), RPS27A (*q* = 0.012784), and RPL18 (*q* = 0.041199), while the following were downregulated: PTPRN2 (*q* = 0.017284), RPS20 (*q* = 4.63E−05), KLC2 (*q* = 0.008854), RPS2 (*q* = 1.02E−06), EEF1A2 (*q* = 1.46E−08), N-R5S113 (*q* = 0.02546), N-R5S111 (*q* = 0.045153), and N-R5S121 (*q* = 0.02531) (Table 5). Protein network analysis of ME8 indicated the involvement of different ribosomal proteins, with high scores, including RPl18, Rps10, Rpl30, Rpl27a, Gm7536, Rps2, Rps13, Rpl9-ps6, Eef1a2, Rps27a, Rpl10, Rps20, and Rpsa (Fig. 4B, C).

**Table 5:** GO (biological process) and KEGG pathway over representation analysis of ME8 module and list of involved genes in wild-type and IL-1b KO mice exposed to acrylamide at 0, 12.5, 25 mg/kg for 28 days by oral gavage

Gene	Regulation	*q* value	Fold change	Wild-type (mg/kg) (mean ± SD)			IL-1b KO (mg/kg) (mean ± SD)			GO/KEGG term
				0	12.5	25	0	12.5	25	
MACF1	Upregulated	1.27E−20	1.951	270 ± 34	242 ± 8	266 ± 14	253 ± 23	528 ± 63	365 ± 13	GO-CC: 0005737-cytoplasm
YWHAH	Upregulated	5.08E−05	1.537	4874 ± 2777	5990 ± 851	6335 ± 937	6201 ± 487	7494 ± 1116	6304 ± 933	GO-CC: 0045202-synapse, GO-CC: 0098793-presynapse,GO-CC: 0005829-cytosol
MT2	Upregulated	0.00586	1.287	490 ± 115	608 ± 131	632 ± 106	451 ± 86	519 ± 137	546 ± 78	GO-CC: 0005829-cytosol, GO-CC: 0005737-cytoplasm
RPS27A	Upregulated	0.012784	1.163	2972 ± 314	3458 ± 257	3353 ± 967	2687 ± 280	2617 ± 531	2908 ± 575	KEGG-mmu03010: Ribosome, GO-CC: 0022626-cytosolic ribosome, GO-CC: 0005840-ribosome, GO-CC: 0022627-cytosolic small ribosomal subunit, GO-CC: 0045202-synapse, GO-CC: 0098794-postsynapse, GO-CC: 0015935-small ribosomal subunit, GO-MF: 0003735-structural constituent of ribosome, GO-BP: 0002181-cytoplasmic translation, GO-BP: 0006412-translation
RPL18	Upregulated	0.041199	1.152	1185 ± 57	1366 ± 104	1276 ± 243	1110 ± 137	1045 ± 164	1215 ± 150	KEGG-mmu03010: Ribosome, KEGG- mmu05171: Coronavirus disease—COVID-19, GO-CC: 0022626-cytosolic ribosome, GO-CC: 0005840-ribosome, GO-CC: 0045202-synapse, GO-CC: 0022625-cytosolic large ribosomal subunit, GO-CC: 0015935-small ribosomal subunit, GO-CC: 0005737-cytoplasm, GO-CC: 0005829-cytosol, GO-CC: 0042788-polysomal ribosome, GO-MF: 0003735-structural constituent of ribosome, GO-BP: 0002181-cytoplasmic translation, GO-BP: 0006412-translation
PTPRN2	Downregulated	0.017284	0.715	171 ± 28	171 ± 14	152 ± 20	142 ± 22	122 ± 21	143 ± 30	GO-CC: 0045202-synapse, GO-CC: 0098793-presynapse,GO-CC: 0005737-cytoplasm
RPS20	Downregulated	4.63E−05	0.706	1566 ± 220	1647 ± 176	1497 ± 419	1306 ± 112	1106 ± 200	1269 ± 222	KEGG-mmu03010: Ribosome, KEGG-mmu05171: Coronavirus disease—COVID-19, GO-CC: 0022626-cytosolic ribosome, GO-CC: 0005840-ribosome, GO-CC: 0022627-cytosolic small ribosomal subunit, GO-CC: 0045202-synapse, GO-CC: 0098794-postsynapse, GO-CC: 0015935-small ribosomal subunit, GO-MF: 0003735-structural constituent of ribosome, GO-BP: 0002181-cytoplasmic translation, GO-BP: 0006412-translation
KLC2	Downregulated	0.008854	0.679	247 ± 37	226 ± 20	207 ± 54	190 ± 36	168 ± 35	204 ± 38	GO-CC: 0005829-cytosol
RPS2	Downregulated	1.02E−06	0.672	2142 ± 339	2303 ± 299	2155 ± 324	2203 ± 290	1441 ± 216	1935 ± 363	KEGG-mmu03010: Ribosome, KEGG-mmu05171: Coronavirus disease—COVID-19, GO-CC: 0022626-cytosolic ribosome, GO-CC: 0005840-ribosome, GO-CC: 0045202-synapse, GO-CC: 0015935-small ribosomal subunit, GO-CC: 0005829-cytosol, GO-MF: 0003735-structural constituent of ribosome, GO-BP: 0002181-cytoplasmic translation, GO-BP: 0006412-translation
EEF1A2	Downregulated	1.46E−08	0.568	528 ± 64	483 ± 67	422 ± 91	361 ± 64	300 ± 44	401 ± 88	GO-CC: 0045202-synaps, GO-CC: 0005737-cytoplasm
N-R5S113	Downregulated	0.02546	0.318	10 ± 4.0	8.9 ± 3.6	8.2 ± 1.8	3.2 ± 1.4	3.2 ± 3.2	6.7 ± 7.3	KEGG-mmu03010: Ribosome, KEGG-mmu03008: Ribosome biogenesis in eukaryotes
N-R5S111	Downregulated	0.045153	0.31	14.6 ± 4.6	13 ± 6.9	11.3 ± 7.7	8.4 ± 2.8	5.2 ± 3.2	7.1 ± 4.7	KEGG-mmu03010: Ribosome, KEGG-mmu03008: Ribosome biogenesis in eukaryotes
N-R5S121	Downregulated	0.02531	0.316	15.8 ± 4	10.6 ± 4.6	11.0 ± 4.9	6.4 ± 2.8	5 ± 4.3	9.4 ± 8.3	KEGG-mmu03010: Ribosome, KEGG-mmu03008: Ribosome biogenesis in eukaryotes

DAVID software was used for analysis. Data of normalized values for gene expression are mean ± SD, *n* = 6. All *P* values for the gene expression were adjusted using Benjamini–Hochberg method and expressed as q values. Fold change represents the ratio of the mean of any group to the mean of the 0 mg/kg wild-type group when the absolute value of logarithm of the ratio is the maximum

*MACF1* microtubule actin crosslinking factor 1, *YWHAH* tyrosine 3-monooxygenase/tryptophan 5-monooxygenase activation protein eta, *MT2* metallothionein 2, *RPS27A* ribosomal protein S27a, *RPL18* ribosomal protein l18, *PTPRN2* protein tyrosine phosphatase receptor type N2, *RPS20* ribosomal protein S20, *KLC2* kinesin light chain 2, *RPS2* ribosomal protein S2, *EEF1A2* elongation factor 1-alpha 2, *n-R5s113* nuclear-encoded rRNA 5S 113, *n-R5s111* nuclear-encoded rRNA 5S 111, *N-R5S121* nuclear-encoded rRNA 5S 121

## Discussion

### IL-1b deletion activates genes in extracellular regions

Transcriptome analysis of ME3 demonstrated upregulation of genes by *IL-1β* deletion in the GO-extracellular regions, including SNORC, PFN1, CRHBP, and PAMR1 (Table 2). The function of SNORC in the central nervous system is unknown at present, but it is highly expressed in the white matter, spinal cord and medulla oblongata and in astrocytes as well as in excitatory neurons at a single cell level (https://www.proteinatlas.org/). A recent study involving transcriptome analysis reported the expression of SNORC in the brain of control subjects but not in cases with Alzheimer disease (Chen et al. 2022). On the other hand, PFN1 (Profilin) gene was reported to be involved in amyotrophic lateral sclerosis (ALS) (Henty-Ridilla et al. 2017). PFN1, which is expressed in almost all eukaryotic cells, was the first actin-binding protein identified to regulate actin dynamics (Zhao et al. 2018). PFN1 is involved in various cellular physiological processes, such as autophagy (Lu et al. 2018) and apoptosis (Yang et al. 2017), as well as in oxidative stress (Li et al. 2018). Knockdown of PFN1 inhibited M1 microglial polarization and promoted M2 microglia polarization 48 h after OGDR stimulation in BV2 cells (Lu et al. 2020). Knockdown of PFN1 also significantly attenuated brain infarcts and edema, improved cerebral blood flow and neurological deficits in MCAO-injured mice (Lu et al. 2020).

The CRHBP gene is linked to the stress pathway, which has been associated with the development of several substance use disorders (SUDs), relapse susceptibility (Levran et al. 2014, 2018), and major depressive disorders (O'Connell et al. 2018). CRH-BP codes for a high affinity binding protein for corticotrophin releasing hormone (CRH), the primary mediator of the mammalian neuroendocrine and behavioral response to stress (Chan et al. 2000). CRH-BP modulates CRH, which influences cortical and hippocampal EEG activity and is the primary mediator of the neuroendocrine stress response. In humans, it has been mapped in several brain regions, including the cerebral cortex (Wang et al. 2007). Thus, the CRHBP is considered candidate gene for anxiety and addiction and possibly required in NMDAR-mediated excitatory postsynaptic currents in the VTA area (Ungless et al. 2003). PAMR1 is known to be downregulated in the hippocampi of homozygous 3xTg AD mouse (Hokama et al. 2014). The MYOC gene is expressed and secreted by optic nerve astrocytes and differentiation of optic nerve oligodendrocytes is delayed in *Myocilin*-null mice. Optic nerves of *Myocilin*-null mice contain low levels of several myelin-associated proteins, including myelin basic protein, myelin proteolipid protein, and 2′3′-cyclic nucleotide 3′-phosphodiesterase, compared with those of wild-type littermates (Kwon et al. 2014). LY86 is mainly expressed in microglia in the central nervous system (protein atlas). In a genomic-wide-association study, alteration of the LY86 gene was noted in the *App*^NL−G−F/NL−G−F^ cortex, suggesting it is a risk factor for AD, as identified by genetic nodes in late-onset AD (Castillo et al. 2017).

A recent study involving protein network analysis in ME3 showed the involvement of WDFY1 protein in a mouse model of schizophrenia (Sancho-Balsells et al. 2020). Furthermore, the same group also described accumulation of WDFY1 protein in the CA1 area of the hippocampus and in the dorsolateral prefrontal cortex in postmortem samples from schizophrenic patients, but not in AD (Sancho-Balsells et al. 2020). Tmem254a is a transmembrane protein known to be expressed in various body tissues, including the brain (https://www.ncbi.nlm.nih.gov) and its expression has been identified in 4 of 18 independent BioProjects that assessed the effects of different stressors on the brain transcriptome in mice (Flati et al. 2020).

The antisense oligonucleotide is a single-strand RNA complementary to a protein coding (mRNA) with which it hybridizes and blocks its translation into protein. The primary function of asRNA is the regulation of gene expression at multiple levels, including transcription, post-transcription, and epigenetic modification (Pelechano and Steinmetz 2013; Wahlestedt 2013; Weiss et al. 1999). Previous studies showed that overexpression of antisense IL-1β transcript suppressed IL-1β expression, suggesting that the antisense-transcripts of innate-immunity-related genes play a role by regulating cytokine expression (Lu et al. 2013). However, the physiological role of IL-1bos remains obscure, but it may play a role in stabilization of the IL-1β KO mice model or in development.

### Mitochondrial oxidative phosphorylation

Several mitochondrial proteins (NADH: ubiquinone oxidoreductase, Ndufs5, Ndufs6, Ndufa7, Ndufa5, Ndufa1, Ndufc1) detected in the present study by the protein network analysis of ME4 and ME7 are known to play important roles in oxidative stress-related processes (Figs. 2B, C, 3B, C). Inefficient oxidative phosphorylation may result in the generation of reactive oxygen species (ROS), leading to mitochondrial dysfunction and worsening of the oxidative stress process (Singh et al. 2019). The upregulated genes identified by KEGG analysis of ME4 (NDUFB8, MT-ATP8, NDUFA12, DNAH7A, MT-CYTB) are also related to mitochondria oxidative phosphorylation process, in addition to the proteasome-related genes PSMB6 and PSMB3 (Table 3). These genes have been reported to be involved in various neurodegenerative diseases, such as Parkinson disease, amyotrophic lateral sclerosis, Huntington disease, Pathways of neurodegeneration-multiple diseases (Table 3). GO and KEGG analyses of ME7 also identified upregulation of genes related to mitochondrial oxidative phosphorylation, including NDUFS6, NDUFA13, NDUFB7, NDUFA3, and NDUFS5, which are involved in a variety of neurodegenerative diseases (Table 4). Complex I (CI or NADH: ubiquinone oxidoreductase) is the largest ETC enzyme of the mitochondria, containing 44 subunits, and the main contributor to ROS production and functional impairments in CI, and seems to be correlated to increased oxidative stress caused by defects in the OXPHOS system (Giachin et al. 2016). Mitochondrial dysfunction represents a common pathogenic mechanism in NDs like Alzheimer’s disease (AD), Parkinson’s disease (PD), ALS, Huntington’s disease, and prion diseases (Lin and Beal 2006; Tillement et al. 2011; Federico et al. 2012; Schapira 2012; Butterfield et al. 2016). Additional complex I deficiency clinical phenotypes have also been associated with pediatric neurodegenerative diseases, including Leigh-like syndrome (LS), leukoencephalopathy, MELAS and NARP syndromes (Giachin et al. 2016). Mutations in 6 mtDNA-encoded (ND1 to 6) and 13 nuclear-encoded (NDUFS1 to 8; NDUFV1; NDUFA1, 2, 9, 10, and 12) CI subunits are considered to be correlated with LS (Rodenburg 2016). NDUFB8 is a signature of the mitochondrial complex I subunit, which is vital for normal mitochondrial function, but when present at high levels, it leads to cellular dysfunction (Davis et al. 2010). On the other hand, mutation of the NDUFA13 subunit leads to instability of mitochondrial complex I that affects motor nerve control by the brain (Angebault et al. 2015). Various in vivo and in vitro models of ALS and patient tissues have confirmed the role of mitochondrial dysfunction in various diseases (Gautam et al. 2019; Nakaya and Maragkakis 2018). This is because motor neurons depend on optimal mitochondrial function to fulfill their energy requirements. Mitochondrial damage causes insufficient ATP production and mediates motor neuron intraneuronal damage and even neuronal death, mediated through high calcium-induced excitotoxicity, increased ROS generation, and activation of the intrinsic apoptotic pathway (Manfredi and Xu 2005; Yakes and VanHouten 1997).

Mitochondrial DNA mutations in the MT-CYTB gene have been detected in the substantia nigra pars compacta and the frontal cortices of patients with PD, compared to the control individuals (Coxhead et al. 2016). ATPase 8 is one of the subunits of the mitochondrial ATP synthase complex (MT-ATP8) and this enzyme is responsible for most of ATP production in the cells (Senior et al. 2002). Several mutations affecting MT-ATP8 have been described in patients presenting with heterogeneous clinical features, varying from neurological to cardiac disorders (Dautant et al. 2018). Overall, mitochondrial respiratory deficiencies have been observed in numerous neurodegenerative disorders, such as AD and PD (Schon and Przedborski 2011). The mitochondrial defects are instrumental in provoking neuronal death in common adult-onset neurodegenerative disorders (Area-Gomez et al. 2019).

### Proteasome

Changes in proteasome activity are closely associated with various neurological conditions, such as AD, PD, HD, and ALS (Im and Chung 2016). Our protein network results showed the involvement of various proteasomes subunits in ME4 in ACR neurotoxicity (Fig. 2B, C).

### Ribosomal proteins

Alteration of ribosomal protein have been reported in a mouse model of frontotemporal dementia (FTD) (Evans et al. 2021). The present study also found changes in the expression of various ribosomal proteins (Fig. 4B, C) and association among ribosomal proteins, as shown by protein network analysis of modules 3 and 8 (Fig. 1C).

### Synapse-related genes

Our results showed significant downregulation of DUSP1, MT-ND3 in ME4 (Table 3). The DUSP1 gene plays a role in the regulation of synaptic plasticity and neuronal morphology, and impairment of the physiological function of DUSP1 has been documented in AD, including the presence of low levels in cortical tissues of AD patients and correlation of these levels with tau pathology and cognitive decline (Perez-Sen et al. 2019). Downregulation in DUSP1 transcripts was also described in the cortex and striatum of mice models of Huntington’s disease (HD) and postmortem samples of HD patients (Luthi-Carter et al. 2002; Taylor et al. 2013), in addition to decrease in DUSP1 activity in PD patients (Collins et al. 2013). Other studies suggested that mitochondrial ND3 (MT-ND3) Aβ interaction could explain, at least in part, the low activity of Complex I in astrocytes and neurons in AD patients (Cruz-Rivera et al. 2018).

In module ME8, ACR exposure significantly upregulated YWHAH, which is involved in synapse pathway (Table 5). The YWHA gene family plays important roles in neuronal and synaptic development, function, and plasticity (Berg et al. 2003; Cornell and Toyo-Oka 2017; Toyo-oka et al. 2003). These genes also participate in the activation of tyrosine and tryptophan hydroxylases, the rate limiting enzymes in the synthesis of certain neurotransmitters, including serotonin and dopamine (Ichimura et al. 1987). Pathologically, evidence suggests that dysregulation of YWHA gene family is involved in the early stages of psychosis (Demars et al. 2020). In our study, the observed upregulation of YWHAH gene suggests its potential role in ACR-induced neurotoxicity and synaptic disfunction in mice. Another gene, Rpl18, is reported to be upregulated in hippocampal lysates of APP/PS1 mice model of SD (Elder et al. 2021). Furthermore, dysregulation of Rpl18 gene expression occurs early in the AD process (Martinez-Ballesteros et al. 2017). Other genes identified in our study, RPS20 and EEF1A, were reported to be downregulated in AD patients (Garcia-Esparcia et al. 2017).

### Genes involved in learning and memory

Humans exposed to electrophiles, such as ACR and 1-bromopropane showed cognitive dysfunction (Ichihara et al. 2002; Igisu et al. 1975). In this regard, protein network analysis in ME4 showed changes in genes known to be involved in learning and memory proteins, such as Egr1, Egr2, Fos, Nr4a1, Btg2 (Barros et al. 2015; Bonow et al. 2009; Poirier et al. 2007; Suzuki et al. 2021; Hawk and Abel 2011) (Fig. 2B, C).

Our recent study demonstrated that exposure to ACR upregulated expression of *Gpx1*, *Gpx4* and *Gclc* in wild-type mice but downregulated them in IL-1β knockout mice (Fergany et al. 2023). In our transcriptome study, the *Gpx4* and *Gclm* show ACR-induced increase in expression in wild-type, but such increase disappeared inIL-1b KO mice. The expression of *Gstm1* and *Gstt2* was altered by ACR exposure similarly in both wild-type and IL-1b KO mice (Supplementary Fig. 1) and there is no ACR-related alteration in expression of *Gpx1* and *Gclc* in both genotypes. These results are partially in consistence with result of (Fergany et al. 2023) which explain the protective effect of IL-1b against acrylamide-induced neurotoxicity.

In conclusions, we have demonstrated in the present study that IL-1b deletion, which potentiates the neurotoxicity of ACR, altered the expression of genes involved in the extracellular region. The results also showed that exposure of mice to ACR or IL-1b deletion altered the expression of genes involved in mitochondrial oxidative phosphorylation, proteasome, ribosome, synapse, learning, and memory, which have been described to be involved in the pathology of various neurodegenerative diseases.

### Supplementary Information

Below is the link to the electronic supplementary material.

Supplementary file1 (DOCX 130 KB)

Supplementary file1 (DOCX 71 KB)

## Data Availability

The raw data generated from the experiment have been deposited in the NCBI Gene Expression Omnibus (GEO, http://www.ncbi.nlm.nih.gov/geo), gene bank accession number (GSE211746).

## Abbreviations

ACR: Acrylamide
AD: Alzheimer disease
ALS: Amyotrophic lateral sclerosis
EEG: Electroencephalogram
FTD: Frontotemporal dementia
HD: Huntington’s disease
MACO: Middle cerebral artery occlusion
MELAS syndrome: Mitochondrial encephalopathy, lactic acidosis, and stroke-like episodes
NARP syndrome: Neuropathy, ataxia, and retinitis pigmentosa
NDs: Neurodegenerative diseases
PD: Parkinson’s disease
ROS: Reactive oxygen species
sRNA: Antisense oligonucleotide
VTA: Ventral trigeminal area
