# Supplementary material for: Transcriptome analysis of the cerebral cortex of acrylamide-exposed wild-type and IL-1β-knockout mice
Source: Arch Toxicol. 2023 Nov 16;98(1):181–205. doi: 10.1007/s00204-023-03627-9 (PMC10761544; doi:10.1007/s00204-023-03627-9)
Supplement: Supplementary file 1 — Supplementary file1 (DOCX 130 KB) [file 204_2023_3627_MOESM1_ESM.docx]

Supplementary Figure 1


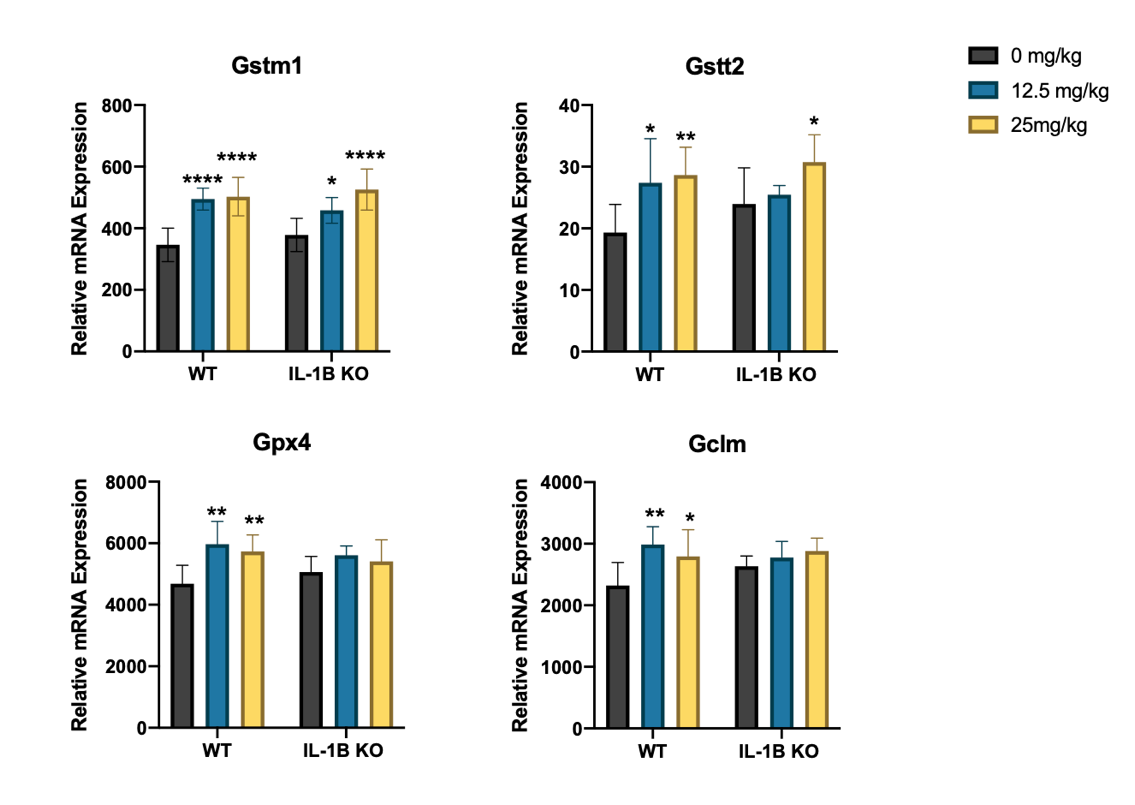


Supplementary Figure1: Changes in mRNA expression levels of Gstm1, Gstt2*, Gpx4, Gclm* in the cerebral cortex of wild-type and *IL-1β* KO mice exposed to acrylamide at 0, 12.5, 25 mg/kg bw for 28 days following transcriptome analysis. Data are individual value plots and bars for mean±SD (n=6 per group). Statistical analyses by ANOVA followed by Dunnett’s multiple comparison test. *P< 0.05, **P<0.01, ***P<0.001, compared to the corresponding genotype control (by Dunnett’s multiple comparison following ANOVA).
