# Supplementary material for: Transcriptome analysis of the cerebral cortex of acrylamide-exposed wild-type and IL-1β-knockout mice
Source: Arch Toxicol. 2023 Nov 16;98(1):181–205. doi: 10.1007/s00204-023-03627-9 (PMC10761544; doi:10.1007/s00204-023-03627-9)
Supplement: Supplementary file 2 — Supplementary file1 (DOCX 71 KB) [file 204_2023_3627_MOESM2_ESM.docx]

Supplementary Table 1: Top 10 upregulated and downregulated genes in ME3 by using the JMP software.

| Gene ID | Fold change | q-value |
| --- | --- | --- |
| Rps15a-ps8 | 65.23604504 | 3.87E-43 |
| Gm26983 | 33.80264387 | 6.03E-19 |
| Myl1 | 21.81180601 | 1.01E-41 |
| Gm5823 | 11.06613305 | 3.82E-56 |
| AI429214 | 6.637728364 | 1.54E-28 |
| Gm13375 | 5.162073769 | 1.56E-04 |
| Snorc | 4.608946319 | 2.05E-07 |
| Il1bos | 3.390088971 | 3.96E-21 |
| Wdfy1 | 3.197975492 | 2.62E-51 |
| Gm10086 | 3.091637735 | 1.16E-17 |
| Rpl34-ps2 | 2.906855903 | 1.85E-31 |
| Ly86 | 0.435974621 | 5.84E-27 |
| Gm8995 | 0.402889193 | 1.04E-01 |
| Ehd1 | 0.365533933 | 9.58E-22 |
| Gpr150 | 0.348751012 | 5.80E-12 |
| Tctex1d2 | 0.337597244 | 1.50E-51 |
| Etnppl | 0.322705866 | 1.77E-19 |
| Gm15503 | 0.308542332 | 6.04E-10 |
| Trim12a | 0.213218799 | 3.51E-07 |
| 2610507I01Rik | 0.044575679 | 1.58E-49 |
| B230311B06Rik | 0.033046687 | 1.55E-31 |

Supplementary Table 2: Top 10 upregulated and downregulated genes in ME4 by using the JMP software

| Gene ID | Fold change | q-value |
| --- | --- | --- |
| Cfap126 | 3.8057396 | 2.69E-03 |
| Gm5914 | 2.42424351 | 6.50E-07 |
| Lyz2 | 2.16238521 | 7.65E-15 |
| Emp3 | 2.13712861 | 2.73E-03 |
| 5830454E08Rik | 2.13022415 | 2.37E-02 |
| A330070K13Rik | 2.12363767 | 1.97E-02 |
| Fam189a2 | 2.10823096 | 8.23E-02 |
| Fgfbp1 | 2.10268479 | 3.08E-03 |
| Gm16437 | 2.01534238 | 3.55E-02 |
| Chrac1 | 1.99263602 | 6.58E-12 |
| Trib1 | 0.44411928 | 1.82E-05 |
| Ccn1 | 0.42038564 | 1.67E-02 |
| Arc | 0.41589481 | 2.34E-06 |
| Gpr39 | 0.41554272 | 1.67E-01 |
| Eya3 | 0.40035482 | 3.63E-08 |
| Rcbtb2 | 0.37889687 | 3.67E-22 |
| Olfr1564 | 0.37612752 | 9.47E-03 |
| Gm10874 | 0.3416111 | 4.06E-03 |
| Six3 | 0.33625266 | 9.74E-02 |
| Slc3a1 | 0.22305442 | 3.27E-06 |

Supplementary Table 3: Top 10 upregulated and downregulated genes in ME7 by using JMP software

| Gene ID | Fold change | q-value |
| --- | --- | --- |
| 1700012B09Rik | 6.09702876 | 2.11E-04 |
| Apoa1 | 2.57835536 | 1.11E-02 |
| Gm5449 | 2.45811853 | 1.34E-01 |
| Atoh7 | 2.4488882 | 3.28E-06 |
| Cebpzos | 2.4098063 | 1.10E-22 |
| Wfdc17 | 2.39852519 | 3.09E-02 |
| 2410080I02Rik | 2.3719547 | 1.16E-02 |
| Agrp | 2.32920509 | 9.69E-03 |
| Xlr3c | 2.17181687 | 1.91E-01 |
| Gm10146 | 2.04155216 | 5.06E-10 |
| Gm43847 | 0.41208916 | 1.48E-02 |
| Gm43118 | 0.36648992 | 2.41E-02 |
| Slc5a7 | 0.36100695 | 2.39E-03 |
| Gm16147 | 0.35521163 | 7.10E-02 |
| Olfr1440 | 0.33796839 | 3.04E-03 |
| Gm19689 | 0.29780787 | 9.70E-06 |
| Atcayos | 0.28372249 | 4.84E-05 |
| Rtl1 | 0.27359722 | 8.85E-03 |
| Cdr1 | 0.2693774 | 1.30E-27 |
| Klhl34 | 0.24923685 | 1.62E-07 |

Supplementary Table 4: Top 10 upregulated and downregulated genes in ME8 by using JMP software

| Gene ID | Fold change | q-value |
| --- | --- | --- |
| Macf1 | 1.951445 | 1.27E-20 |
| Xkr6 | 1.87819611 | 6.98E-03 |
| Gm11448 | 1.75159535 | 6.91E-02 |
| Hmgb1-ps4 | 1.5831561 | 2.49E-01 |
| Gm4575 | 1.57056431 | 2.94E-01 |
| Ywhah | 1.53746067 | 5.08E-05 |
| Zfp53 | 1.52200023 | 7.11E-02 |
| Gm12428 | 1.51440107 | 9.57E-03 |
| Haus3 | 1.41824087 | 1.26E-01 |
| 2610316D01Rik | 1.41772729 | 2.20E-01 |
| n-R5s138 | 0.36099967 | 3.02E-01 |
| n-R5s111 | 0.3560803 | 4.52E-02 |
| n-R5s144 | 0.35245139 | 8.11E-03 |
| n-R5s143 | 0.34424557 | 8.77E-03 |
| Gm25018 | 0.32863098 | 1.09E-01 |
| n-R5s113 | 0.31858241 | 2.55E-02 |
| n-R5s121 | 0.31673997 | 2.53E-02 |
| n-R5s128 | 0.25918798 | 1.96E-01 |
